# Current Development of siRNA Bioconjugates: From Research to the Clinic

**DOI:** 10.3389/fphar.2019.00444

**Published:** 2019-04-26

**Authors:** Ivan V. Chernikov, Valentin V. Vlassov, Elena L. Chernolovskaya

**Affiliations:** Laboratory of Nucleic Acids Biochemistry, Institute of Chemical Biology and Fundamental Medicine, Siberian Branch of the Russian Academy of Sciences, Novosibirsk, Russia

**Keywords:** RNAi, siRNA, bioconjugate, chemical modifications, patterns of chemical modifications

## Abstract

Small interfering RNAs (siRNAs) acting via RNA interference mechanisms are able to recognize a homologous mRNA sequence in the cell and induce its degradation. The main problems in the development of siRNA-based drugs for therapeutic use are the low efficiency of siRNA delivery to target cells and the degradation of siRNAs by nucleases in biological fluids. Various approaches have been proposed to solve the problem of siRNA delivery *in vivo* (e.g., viruses, cationic lipids, polymers, nanoparticles), but all have limitations for therapeutic use. One of the most promising approaches to solve the problem of siRNA delivery to target cells is bioconjugation; i.e., the covalent connection of siRNAs with biogenic molecules (lipophilic molecules, antibodies, aptamers, ligands, peptides, or polymers). Bioconjugates are “ideal nanoparticles” since they do not need a positive charge to form complexes, are less toxic, and are less effectively recognized by components of the immune system because of their small size. This review is focused on strategies and principles for constructing siRNA bioconjugates for *in vivo* use.

## Introduction

Small interfering RNAs (siRNAs) are the most promising type of RNA-based therapeutic oligonucleotide drug, since their mechanism of action is catalytic and each siRNA molecule can inactivate several target RNA molecules in a sequence-specific manner. Since the discovery of RNA interference (RNAi) and the development of the first oligomeric RNAs that trigger RNAi in mammalian cells, significant progress has been made in the development of therapeutic siRNAs (Fire et al., 1998; Crooke et al., 2018). Chemical modifications of RNA have been developed that modulate their activity and stabilize them in biological fluids (Hoerter and Walter, 2007); some progress has been made in the development of methods for the delivery of siRNAs to cells (Hassler et al., 2018). Several siRNA-based drugs are undergoing clinical trials, and one drug patisiran (Onpattro) is approved for use in the clinic (Garber, 2018). However, the outstanding potential of siRNAs as therapeutic drugs has not yet been fully implemented. A number of unsolved problems remain: it is essential to develop an effective means of delivering siRNAs to certain types of cells; it is also necessary to create modified versions of siRNAs that are stable, effectively silence target RNAs, and do not cause side effects. These problems are a consequence of the properties of siRNAs, which are large, polyanionic molecules that are unstable in biological media and are capable of causing unwanted immune responses when they enter cells.

## RNAi

**Figure 1:**
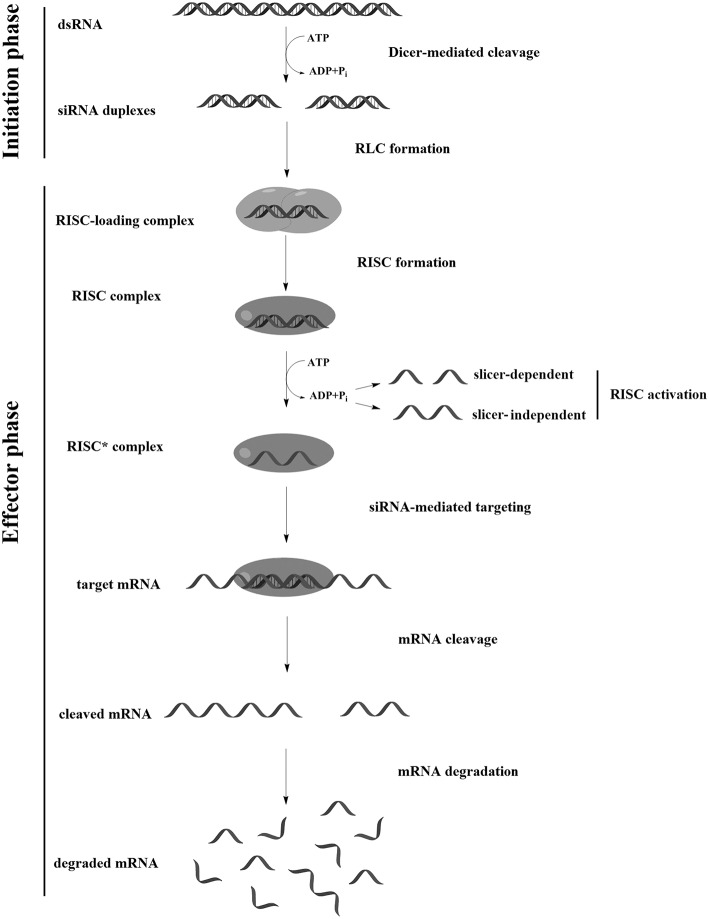
RNA interference (RNAi) mechanism.

Induction of RNAi occurs when double-stranded RNA (dsRNA) enters the cell; e.g., when transfected with dsRNA, infected with RNA-containing viruses (De Paula et al., 2007), or when endogenously formed in cells as a result of transposon or non-coding RNA expression (Khvorova et al., 2003). Mechanism of RNAi is divided in to two phases: in the first stage (initiation phase), long dsRNA is cleaved by the endoribonuclease Dicer into siRNAs, short dsRNAs (21–23 bp) with two nucleotides protruding at the 3′-ends. In the second stage (effector phase), the multiprotein RNA-induced silencing complex (RISC) is formed, which, after activation, performs recognition and sequence-specific cleavage of the target mRNA (Figure 1). It has been shown that RNAi in mammalian cells can be induced by chemically or enzymatically synthesized siRNAs that mimic dsRNA Dicer cleavage products (Nakanishi, 2016); in this case, the RNAi mechanism involves only the effector phase.

In the first stage of RISC assembly, the R2D2 protein (in *Drosophila*) or its analog (in other species), which contains two dsRNA binding domains and the Dicer binding domain, binds the siRNA. R2D2 recognizes and binds the thermodynamically more stable 5′ end of the duplex, which allows further binding of Dicer (Tomari et al., 2004), whose dsRNA-binding domain has specificity for 3′-overhangs (Ma et al., 2004). Thus, the intermediate RISC loading complex (RLC) is formed. Following formation of the RLC, Dicer interacts with argonaute-2 (Ago2), presumably with the participation of the PAZ domains (Bernstein et al., 2001).

At the last stage of RISC assembly, Ago2 cuts and causes dissociation of one of the siRNA strands (“passenger strand”), resulting in the formation of an activated RISC^*^. Ago2 and the remaining siRNA strand (“guide strand”) are the main components of activated RISC (Aronin, 2006; Addepalli et al., 2010); however, a number of other proteins may also be part of this complex (Rana, 2007; Ohrt et al., 2008). The selection of the strand that is included in RISC^*^ is determined by the orientation of the Dicer-R2D2 heterodimer relative to the siRNA; since R2D2 interacts with the thermodynamically more stable end of the duplex, the most active siRNAs are those with a 5′ end of the sense strand more thermostable than the 5′ end of the antisense strand. Ago2 cuts both the siRNA passenger strand and the target mRNA (Liu et al., 2004); however, siRNA strand dissociation can be carried out without cleavage. Moreover, it is assumed that human Ago2 causes strand dissociation, mainly by a mechanism that does not require its cleavage (Muhonen et al., 2007; Park and Shin, 2015), therefore, the total melting point of the duplex can contribute to the efficiency of siRNA interfering activity. Recognition of the mRNA target by RISC^*^ occurs in several stages, wherein the “seed” region (the siRNA region from 2 to 8 nucleotides from the 5′ end of antisense strand) plays an important role. First, an initial screening of the sequence for three nucleotides (2–4 nucleotides from the 5′ end of the siRNA strand) occurs (Chandradoss et al., 2015). After the triplet is recognized, the fifth nucleotide from the 5′ end of the antisense strand interacts with the target mRNA, which contributes to conformational changes, opening nucleotides 6–8 and 13–16 for interaction (Schirle et al., 2014). The complementary interaction of the siRNA strand with the mRNA provides an advantageous conformation to cleave the mRNA between nucleotides 10–11 relative to the 5′ end of the siRNA, which occurs via the PIWI domain of Ago2 (Jinek and Doudna, 2009). After cutting and dissociating from the complex, the target RNA and passenger strand of the siRNA are degraded by ribonucleases. Released RISC^*^ can participate in subsequent cycles of cleavage in a catalytic manner (Haley and Zamore, 2004; Aronin, 2006; Leuschner et al., 2006).

Due to the high affinity of RISC^*^ to single-stranded RNA, binding efficiency of RISC^*^ with the target mRNA is almost an order of magnitude greater than that of antisense oligodeoxynucleotides (ASOs) with the same sequence in which binding to the target mRNA occurs only through complementary interaction (Ameres et al., 2007). Thus, the concentration of siRNA at which an effective decrease in the expression of the target gene is observed is two to three orders of magnitude lower compared to antisense oligonucleotides (Lemaitre et al., 1987; Subramanian et al., 2015).

## Barriers for siRNA to Their Targets

There are a number of biological barriers that impede the effective action of RNA in mammalian cells. First, since siRNAs are polyanions, they are unable to penetrate directly through the hydrophobic cell membrane and can enter the cell only by endocytosis or pinocytosis. However, in order to implement the silencing effect, endocytosed siRNA must penetrate the endosome membrane and exit into the cytoplasm, otherwise it will undergo cleavage by ribonucleases (Varkouhi et al., 2011), or will leave the cell via exocytosis (Shukla et al., 2016). When siRNA enters the cytoplasmic space, it can also be cleaved by cytoplasmic ribonucleases (Whitehead et al., 2009) or its concentration can decrease due to the division of target cells.

Despite the high specificity of the action, some siRNA can cause a number of non-targeted effects that prevent its use in high concentrations due to the toxicity they cause. The most significant non-targeted effect of siRNA is unwanted activation of the system of innate immunity under the action of certain motifs in the siRNA sequence. When interacting with the membrane surface or in the endosome, immunostimulating motifs can be recognized by Toll-like receptors (TLR3/7/8) (Oosenbrug et al., 2017; Pirher et al., 2017), inducing the production of interferons (α or β) and inflammatory cytokines that activate immune response and induce global changes in gene expression pattern (Mansoori et al., 2016). Other non-target effect of siRNAs is the displacement of endogenous micro-RNA from RISC, which can disrupt the natural regulation pathways in the cell. Also, the sense strand can be introduced in RISC^*^ and suppress the expression of non-target genes, similar effects can be caused by the antisense strand of siRNA which bind to partially homologous non-target mRNA. In last case the block of translation does not include the cleavage of mRNA (Huntzinger and Izaurralde, 2011).

In the transition to the level of the organism, there are new factors that reduce the effectiveness of the siRNA, such as: siRNA filtration by the kidneys, siRNA capture by immune cells, cleavage by serum ribonucleases, the endothelial barrier (Kanasty et al., 2012, p. 511). Due to the presence of these barriers, siRNA have reduced bioavailability and unfavorable pharmacokinetics *in vivo*, which necessitates the use of high doses of siRNA and makes it not always possible to achieve the desired effect. This review examines approaches to solving the above problems based on chemical modifications of siRNAs; namely, introducing unnatural nucleotides into the siRNA structure and attaching molecules to siRNAs, ensuring the interaction of conjugates with biological structures that increase the efficiency and specificity of siRNAs as potential drugs.

## Chemical Modifications of siRNA

**Figure 2:**
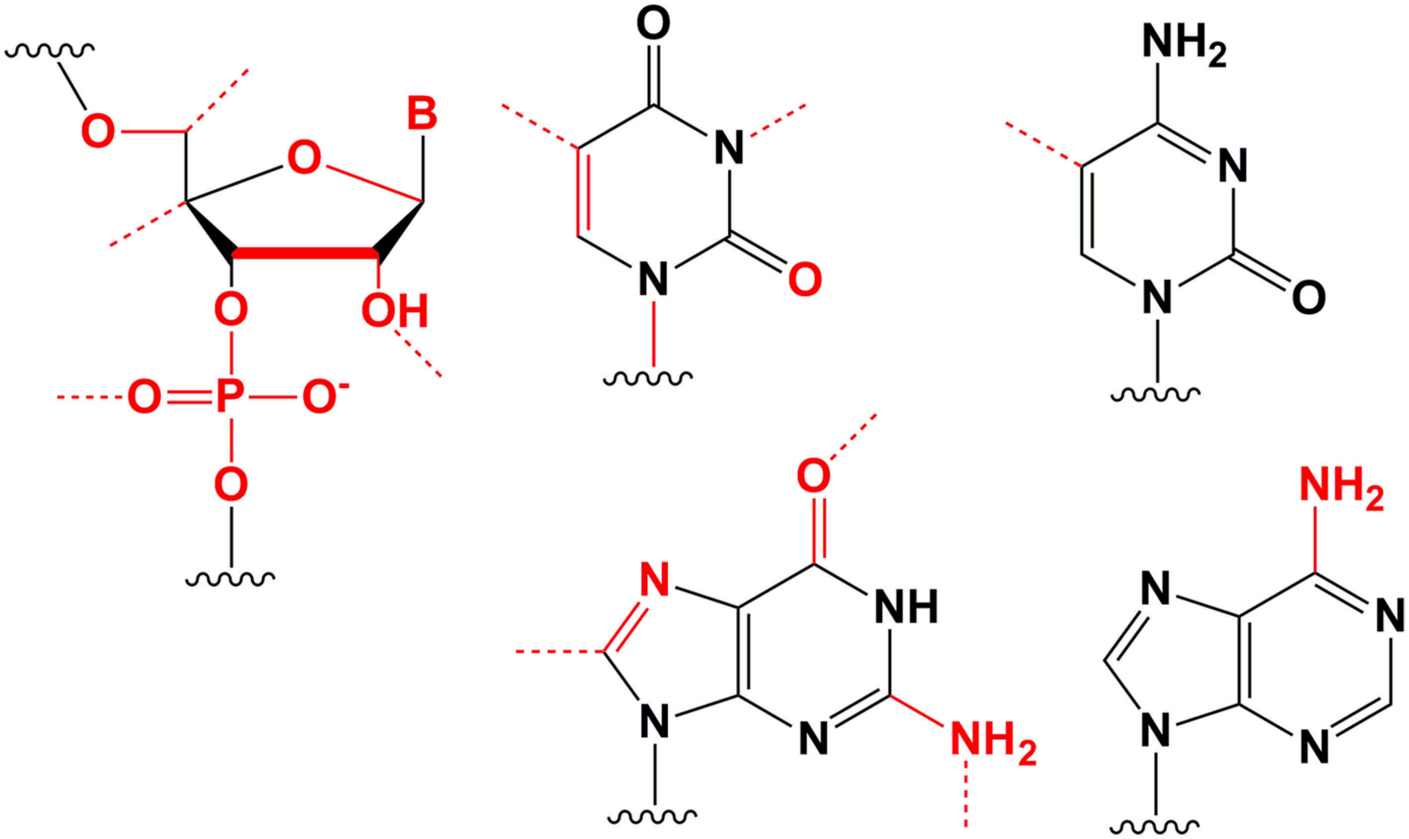
Sites of introduction of chemical modifications in siRNAs (marked in red).

Chemical modifications may affect the properties of siRNA: sensitivity to ribonucleases, recognition by the RNAi system, hydrophobicity, toxicity, duplex melting temperature, and conformation of the RNA helix (Manoharan, 2004; Behlke, 2008; Chen et al., 2018). Modifications can be divided into modifications of ribose, phosphates, and nucleobases (Table 1; Figure 2). Each type of modification is reviewed separately below.

**Table 1:** The effect of chemical modifications on siRNA properties.

**Modification**	**Structure**	**_**Δ**_T_**m**_ duplex per modi-fication**	**Impact on the efficiency of RNAi**	**Other properties of modification (effect on ribose conformation, nuclease resistance, toxicity, etc.)**
**SUGAR MODIFICATIONS**				
2′-O-methyl (2′O-Me)	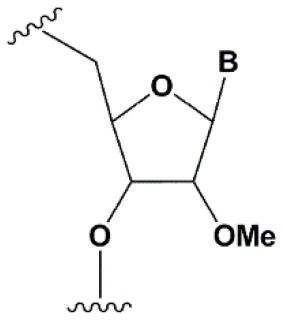	+0.5–1.5°C	Two or more consecutive 2′O-Me inhibits RNAi (Czauderna et al., 2003; Prakash et al., 2005; Akinc et al., 2008; Manoharan et al., 2011). However, siRNAs possessing biological activity, containing 75–82% 2′O-Me, are described (Ray et al., 2017; Foster et al., 2018).	Stabilizes 3′*endo* ribose conformation. ≥5–30% of 2′O-Me increase nuclease resistance *in vitro* (Jackson et al., 2006; Volkov et al., 2009; Petrova Kruglova et al., 2010; Takahashi et al., 2012) and *in vivo* (Liu et al., 2014; Chernikov et al., 2017). 2′O-Me analogs of A, G and U reduce the immune response (Judge et al., 2006).
2′-fluoro (2′F)	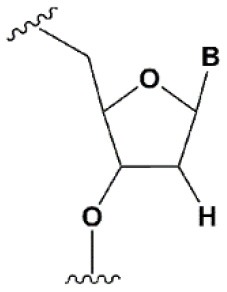	+1.5–4°C	2′F analogs in all siRNA positions only slightly reduces the activity of RNAi (Deleavey et al., 2010).	Stabilizes 3′*endo* ribose conformation. ≥50% 2′F increase nuclease resistance *in vitro* (Cuellar et al., 2014) and *in vivo* (Viel et al., 2008; Manoharan et al., 2011). 2′F analogs of adenine (≥7%) reduce the immune response *in vitro* (Fucini et al., 2012). >50% of the 2′F in siRNA may cause toxicity (Ohrt and Schwille, 2008; Shen et al., 2015; Garber, 2016).
2′F-arabinonucleic acid (2′FANA)	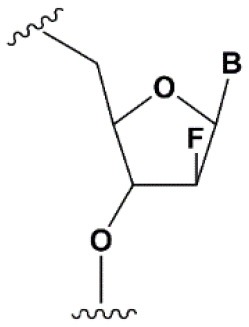	+1.2°C	100% 2′FANA in the sense chain reduce the efficiency of RNAi. ≥30% 2′FANA in the antisense chain inhibits RNAi (Dowler et al., 2006; Deleavey et al., 2010).	Stabilizes 2′*endo* ribose conformation. ≥50% 2′FANA increases nuclease resistance *in vitro* (Deleavey et al., 2010); more effectively than 2′F protect siRNA from the action of exoribonucleases (Damha et al., 2001).
2′-O-methoxyethyl (2′O-MOE)	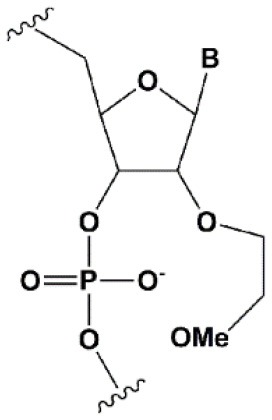	+0.9–1.7°C	2′-MOE at the flanks of the sense strand and the central part (6–11) of the antisense strand are tolerable for RNAi (Prakash et al., 2005; Manoharan et al., 2011). Replacement of 9th or 10th nucleotides from the 5′ end to 2′O-MOE analogs of nucleotide increases the probability of entry in RISC (Song et al., 2017).	Stabilizes 3′*endo* ribose conformation. ≥15% 2′O-MOE at the ends of the siRNA sense chain increases nuclease resistance *in vitro* (Lima et al., 2012).
Locked nucleic acid (LNA)	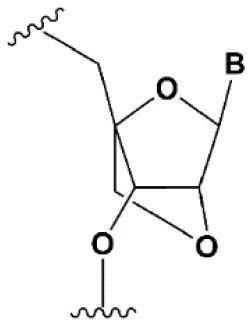	+2–8°C	≥40% LNA in the sense chain inhibit RNAi by 5–20% (Elmen et al., 2005). >20% LNA in the antisense chain, or the first LNA nucleotide at the 5′ end completely inhibit RNA (Braasch et al., 2003; Elmen et al., 2005; Mook et al., 2007; Schyth et al., 2012). LNA can change thermal asymmetry of the duplex, increasing the efficiency of siRNA (Elmen et al., 2005).	Reduces the conformational flexibility of nucleotides, fixing the C3′*endo* conformation of the ribose (Julien et al., 2008). ≥10–20% LNA in siRNAs increase nuclease resistance *in vitro* (Elmen et al., 2005) and *in vivo* (Mook et al., 2010).
Unlocked nucleic acid (UNA)	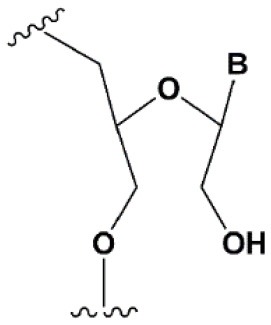	−5–8°C	>15% UNA inhibit RNAi (Laursen et al., 2010). UNA can change thermal asymmetry of the duplex, increasing the efficiency of siRNA (Mook et al., 2010; Vaish et al., 2011; Snead et al., 2013).	Increases conformational flexibility of nucleotides and reduces the melting point of the duplex. UNA at the 3′ends of the duplex protect siRNA from 3′ exoribonucleases *in vitro* and *in vivo* (Laursen et al., 2010; Mook et al., 2010; Pasternak and Wengel, 2011). 4′-thioribonucleosides (4′S)
4′-thioribonucleosides (4′S)	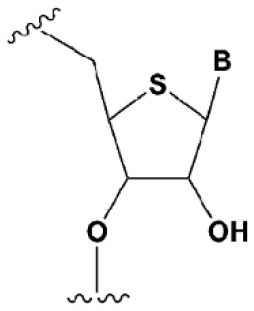	+1°C	>7–15% 4′S in the antisense strand inhibit RNAi (Hoshika et al., 2005, 2007; Dande et al., 2006).	>10–15% 4′S at the ends of the strands increase the nuclease resistance *in vitro* (Dande et al., 2006; Takahashi et al., 2012).
4′-C-aminomethyl-2′-O-methyl	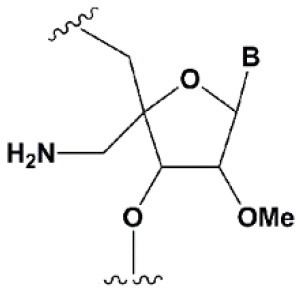	−1°C	>2 analogs in the sense or >1 analog in the antisense strand inhibit RNAi (Gore et al., 2012).	≥2 modifications at the 3′ ends increase nuclease resistance *in vitro* (Gore et al., 2012).
Deoxyribonucleotide (dNMP)	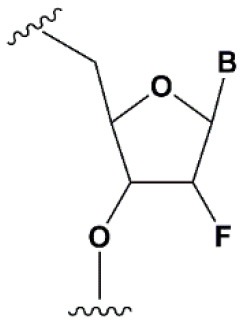	−0.5°C	>50% dNMP inhibits RNAi (Parrish et al., 2000; Elbashir et al., 2001; Ui-Tei et al., 2008). dNMP can change thermal asymmetry of the duplex, increasing the efficiency of siRNA *in vitro* (Ui-Tei et al., 2008).	Protects against exoribonucleases (Parrish et al., 2000).
Cyclohexenyl nucleic acids (CeNA)	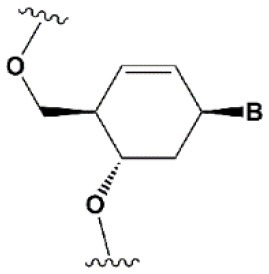	+1.5°C	5% CeNA in siRNA are tolerated by RNAi (Herdewijn and Juliano, 2007; Nauwelaerts et al., 2007). CeNA can change thermal asymmetry of the duplex, increasing the efficiency of siRNA *in vitro* (Herdewijn and Juliano, 2007; Fisher et al., 2009).	Stabilizes 3′*endo* ribose conformation (Ovaere et al., 2011). ≥25% CeNA analogs increase serum nuclease resistance (Wang et al., 2001).
Hexitol nucleic acids (HNA)	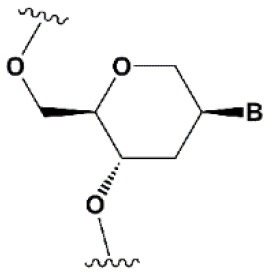	+0.85°C	15% HNA in siRNA are tolerated by RNAi (Fisher et al., 2009). HNA can change thermal asymmetry of the duplex, increasing the efficiency of siRNA *in vitro* (Herdewijn and Juliano, 2007).	Slightly increases siRNA resistance to nucleases in serum (Fisher et al., 2009).
**PHOSPHATE BACKBONE MODIFICATIONS**				
Phosphorothioate (PS)	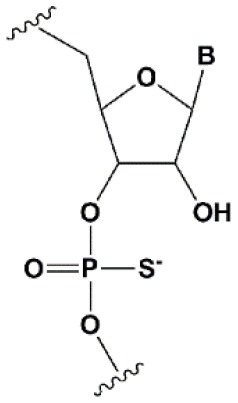	−0.7°C	PS inhibits RNAi when introduced in the central part of the antisense strand (Amarzguioui et al., 2003; Schwarz et al., 2004; Prakash et al., 2005; Eckstein, 2014).	PS protects siRNAs from the action of exoribonucleases *in vitro* and *in vivo* (Soutschek et al., 2004). >50% PS cause toxicity *in vitro* (Harborth et al., 2003) and *in vivo* (Henry et al., 2002; Iannitti et al., 2014). Dimethylethylenediamine (DMEDA)
Dimethylethylenediamine (DMEDA)	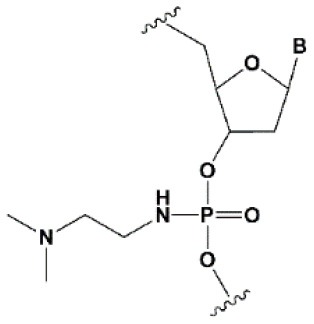	−0.7–3.4°C (shown only for thymidine)	10% DMEDA in the sense strand are tolerated by RNAi (Vlaho et al., 2017).	The effect on nuclease resistance of siRNA was not shown.
*Tert*-butyl-S-acyl-2-thioethyl (*t*Bu-SATE)	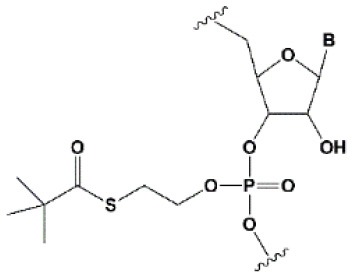	No data.	25% *t*Bu-SATE are tolerated by RNAi (Meade et al., 2014).	≥20–40% *t*Bu-SATE in siRNA increase nuclease resistance *in vitro* and *in vivo* (Meade et al., 2014). Increases hydrophobicity of siRNA. Cleaved by thioesterase in the cytoplasm of the cell giving a phosphodiester bond (Meade et al., 2014).
Boranophosphate (BP)	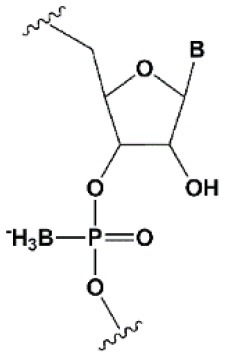	+0.4–1°C (<50% of siRNA) −0.8–2.5°C (>50% of siRNA)	>50% PB inhibit RNAi, the central part of the antisense strand is the most sensitive to modifications (Hall et al., 2004).	Approximately two times more effectively protect against ribonucleases than PS, but do not cause toxicity *in vitro* (Hall et al., 2004, 2006).
Amide linker	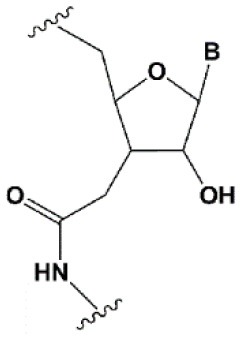	−0.3 to +0.9°C	In some siRNA positions, a single substitution for an amide linker is tolerated by RNAi (Mutisya et al., 2017).	The introduction of two amide linkers from the 3′ ends of the duplex increases the nuclease resistance of siRNA in serum (Iwase et al., 2007; Selvam et al., 2011).
**5′-PHOSPHATE MODIFICATIONS**				
5′C-methyl (*S*-isomer)	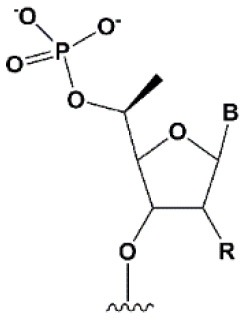	−3.2°C	One (*S*) 5′C-methyl at the 5′ end of the antisense strand is tolerated by RNAi (Prakash et al., 2015).	(*S*) 5′C-methyl protect siRNA from exonucleases two times more efficiently than PS (Kel'in et al., 2016).
5′ (*E*)-vinylphosphonate	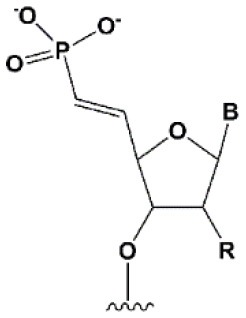	No data.	5′(*E*)-vinylphosphonate 2-folds improves siRNA interaction efficiency with Ago2 (Elkayam et al., 2017). Does not change the biological activity of siRNA *in vitro* (Haraszti et al., 2017).	Stabilizes 5′ phosphate, protect from the action of phosphatases and exonucleases. Improves the pharmacokinetics (Elkayam et al., 2017; Haraszti et al., 2017). 5′ methylenephosphonate
5′ methylenephosphonate	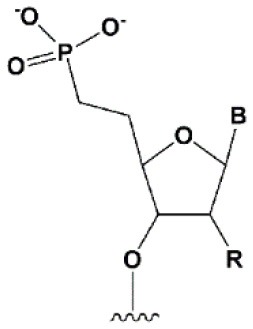	No data.	5′ methylenephosphonate at the 5′ end of the antisense strand reduces the biological activity of siRNA by ~10-folds (Lima et al., 2012; Prakash et al., 2015).	No data.
**BASE MODIFICATIONS**				
2′ thiouridine (s^2^U)	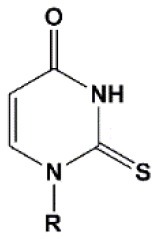	0–2°C	7% s^2^U are tolerated by RNAi (Sipa et al., 2007). s^2^U can change thermal asymmetry of the duplex, increasing the efficiency of siRNA *in vitro* (Sipa et al., 2007; Peacock et al., 2011).	s^2^U slightly increases nuclease resistance *in vitro*.
Pseudouridine (Ψ)	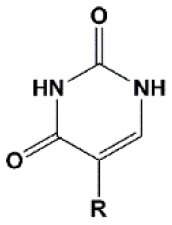	−1 to +1°C	One Ψ is tolerated by RNA(Sipa et al., 2007).	Stabilizes 3′*endo* ribose conformation. Reduces the PKR-induced interferon response (Anderson et al., 2010).

### Ribose Modifications

Among all ribose modifications, substitutions in the 2′-position most effectively protect siRNAs against the action of serum nucleases, as the 2′OH group participates in the cleavage of RNA by endoribonucleases (Findlay et al., 1962; Breslow and Chapman, 1996). In this case, the size of the substituent at the 2′-position of ribose affects the properties of the modified base. When the hydrogen of the 2′OH group is replaced with a relatively small methyl residue (2′-O-methyl modification [2′O-Me]) (Bobst et al., 1969), there is stabilization of the 3′-*endo* ribose conformation, which provides the A-type RNA helix essential for RNAi. The introduction of 2′O-Me modifications into siRNA promotes its protection against nucleases both *in vitro* (Volkov et al., 2009) and *in vivo* (Liu et al., 2014; Chernikov et al., 2017). Moreover, the introduction of these modifications reduces the immune response (Judge et al., 2006). These properties make 2′O-Me modification of siRNA one of the most attractive strategies for introducing siRNA-based drugs into the clinic (Khvorova, 2017; Ray et al., 2017). However, replacement of each nucleotide in the siRNA with a 2′O-Me modified one leads to inhibition of RNAi (Czauderna et al., 2003); even the replacement of 50% of the nucleotides in siRNAs with 2′O-Me could inhibit this process. The presence of a hydrophobic residue at the 2′ position alters the overall structure of siRNA and increases the thermal stability of the duplex, which interferes with its effective incorporation into RISC and dissociation of the passenger strand.

Since the size of the modification may contribute to an increase in the nuclease resistance of siRNA (Cummins et al., 1995), attempts have been made to introduce more voluminous substituents into the 2′ position of ribose (2′-O-methoxyethyl [2′O-MOE] Prakash et al., 2005; Zanardi et al., 2018, 2′O-allyl Amarzguioui et al., 2003, 2′O-benzyl Kenski et al., 2012, and other modifications); however, these substitutions more significantly inhibited RNAi than 2′O-Me.

Among the large number of bulky 2′ substituents, the 2′O-MOE ribose is one of the few modifications that stabilizes the 3′ *endo* ribose conformation and increases the melting temperature of the duplex more effectively than 2′O-Me (Dorn et al., 2004). The introduction of 2′O-MOE into siRNA without inhibiting RNAi is possible only along the flanks of the duplex and the central part of the antisense strand (Prakash et al., 2005). Availability of 2′O-MOE in the central part of the antisense strand (9 or 10 nucleotides) was recently shown to increase the biological activity of siRNA by reducing the probability of inclusion of the sense strand in RISC (Song et al., 2017), although direct confirmation of the mechanism of modified strands selection was not provided. Apparently, the presence of a bulk modification in this position may sterically affect the interaction with a Dicer-R2D2 heterodimer or the formation of RISC (Koller et al., 2006).

Unlike the substitution of hydrogen in the 2′OH group, the replacement of oxygen by 2′-fluorine (2′F) is more consistent with the original structure of RNA, effectively stabilizing the 3′ *endo* ribose conformation (Manoharan et al., 2011). Introduction of 2′F into all nucleotides of the duplex only slightly reduces the efficiency of RNAi (Blidner et al., 2007; Deleavey et al., 2010). This modification protects siRNAs from the action of nucleases *in vitro* and *in vivo* (Manoharan et al., 2011; Fucini et al., 2012); however, the introduction of modifications in 50% of the siRNA nucleotides could lead to toxicity (Shen et al., 2015; Janas et al., 2017). In 2016, the third stage of clinical trials of the Alnylam conjugate of N-acetylgalactosamine and siRNA containing 50% 2′F revealed its cardiotoxicity (Garber, 2016). Since these effects were not detected in previous stages of clinical trials (Zimmermann et al., 2017) and the toxicity of 2′F modified siRNA for the heart was not previously shown, these results may be occasional and not related to the properties of the conjugate. On the other hand, it was shown that under the action of ASO containing 50% 2′F, the expression profiles of a number of genes *in vitro* (Shen et al., 2015) and *in vivo* (Shen et al., 2018) were altered. In confirmation of the toxicity hypothesis of siRNAs containing 2′F, another study showed that the introduction of 2′F at the ends of the duplex alters the localization of siRNA from cytoplasmic to nuclear (Ohrt and Schwille, 2008). These results suggested that only a limited amount (no more than 25%) of 2′F modifications (Ray et al., 2017) could be introduced for therapeutic applications. However, a decrease in the proportion of 2′F analogs from 50 to 25% did not lead to a decrease in hepatotoxicity in rats and mice following intravenous administration of high doses (100–200 mg/kg) of siRNA and N-acetylgalactosamine conjugate (Janas et al., 2018).

Although other positions in ribose, such as 4′ carbon, can be modified [4′S Gore et al., 2012, 4′C-aminomethyl-2′-O-methyl Takahashi et al., 2012, and 4′C-O-methyl-2′-O-methyl Harp et al., 2018 (Table 1)] and such modifications protect siRNAs from nucleases *in vitro* efficiently, these modifications are not widely used in biomedical research because they significantly inhibit RNAi (Deleavey and Damha, 2012).

Ribose modifications are not limited to substitutions in structure; nucleic acid analogs with a modified structure of the furanose cycle, such as derivatives containing 6-membered (hexitol [HNA] Fisher et al., 2009, cyclohexenic [CeNA] Nauwelaerts et al., 2007, and altritol [ANA] Fisher et al., 2007 nucleic acids) and 7-membered rings (oxepanic nucleic acid [ONA] Sabatino and Damha, 2007), bicyclic (locked nucleic acids [LNA] Braasch et al., 2003, 2′-deoxymethanocarbanucleosides [MCs] Terrazas et al., 2011), tricyclic (tricyclo-DNA [tc-DNA] Goyenvalle et al., 2015), and acyclic (unlocked nucleic acid [UNA] Jensen et al., 2008; Langkjaer et al., 2009) derivatives, can protect siRNAs from the action of nucleases and in some cases (CeNA, LNA, and UNA) do not inhibit RNAi (Herdewijn and Juliano, 2007; Deleavey and Damha, 2012). Among the 6-membered nucleic acid derivatives, CeNA is most suitable for modifying siRNA, since its complementary interaction with RNA stabilizes the duplex, increasing the melting point by 1.5°C per modified base and increases the oligoribonucleotide resistance to degradation in serum (Wang et al., 2001). Bicyclic derivatives (LNA) can even more significantly increase the melting temperature of siRNA. In the case of LNA, affinity for the complementary strand is increased by 2–8°C per nucleotide due to the extra cycle between 2′ and 4′ carbon, which fixes the 3′ *endo* ribose conformation (Julien et al., 2008). However, the introduction of this modification into siRNA strongly affects its interfering activity and the antisense strand is especially sensitive to this modification; even one LNA modification of its first nucleotide from the 5′ end completely inhibits RNAi (Elmen et al., 2005). Conversely, conformationally more flexible acyclic derivatives, such as UNA or glycolic nucleic acid (GNA), can destabilize the duplex, reducing the melting point by 5–8 and 5–18°C per nucleotide, respectively (Laursen et al., 2010; Schlegel et al., 2017).

Since thermal asymmetry of the duplex makes a primary contribution to “guide” strand selection, modifications stabilizing the duplex formed by the 3′ end of the antisense strand and 5′ end of the sense strand and, conversely, modifications destabilizing the duplex formed by the 3′ end of the sense strand and 5′ end of the antisense strand can increase the efficiency of RNAi by providing favorable duplex thermal asymmetry. Thus, the introduction of LNA, UNA, or GNA at different ends of the duplex can lead to an increase in siRNA efficiency by increasing the probability of incorporation of the antisense strand into RISC (Vaish et al., 2011). Moreover, due the change in thermal asymmetry of siRNA, the probability of incorporation of the sense strand in RISC and the following suppression of expression of non-target genes those have regions complementary to the sense strand of the siRNA decreases.

The antisense strand of siRNA can also block the translation of non-target mRNA by complementary interaction with the “seed” region. It has been shown that a decrease in the melting temperature of this region leads to a decrease in the efficiency of suppression of non-target genes (Jackson et al., 2006). Therefore, the introduction of UNA or GNA modifications in the “seed” region of the antisense strand of siRNAs contributes to an increase in the specificity of action (Bramsen et al., 2009; Janas et al., 2017).

An interesting strategy to increase the specificity of the siRNA is introducing nicks in the middle of the sense strand of the siRNA (small internally segmented interfering RNA [sisiRNA]) (Bramsen et al., 2007). sisiRNAs have a greater specificity of biological action because RISC containing sense strand is not formed. sisiRNAs are less stable compared to siRNAs of the same sequence; thus, LNA modifications are introduced to stabilize sisiRNAs. However, it has been shown that such a duplex design did not contribute to a significant increase in the biological activity of siRNA *in vitro* (Hong and Nam, 2016) or *in vivo* (Mook et al., 2010).

### Phosphate Modifications

Ribose modifications do not interfere with changes in the phosphate structure, and since that modifications are directly involved in nuclease cleavage (Frazao et al., 2006), it is reasonable that such modifications could effectively protect siRNA from degradation. Replacement of the oxygen of phosphate with sulfur (phosphorothioate [PS] Eckstein, 1970, 2014) or boron (boranophosphate [BP] Hall et al., 2006) has been shown to protect siRNA from the action of ribonucleases *in vitro* and *in vivo* (Soutschek et al., 2004). The introduction of PS modifications to both strands of the duplex inhibits RNAi to some extent (Schwarz et al., 2004). However, the introduction of PS modifications into oligonucleotides facilitates their penetration into cells in the absence of transfection agents due to non-specific binding to cell receptors and penetration by clathrin-dependent endocytosis (Wang et al., 2018). On the other hand, due to the non-specific interaction of PS oligonucleotides with serum proteins and cell receptors (Lee et al., 1999), activation of the complement system and leukocyte infiltration of the corresponding organs (Iannitti et al., 2014) may occur. Therefore, for clinical use of siRNA, the number of PS modifications should be reduced to 5–50%, depending on the intended dose of siRNA.

Unlike PS, the introduction of BP into the central part of the antisense strand strongly inhibits the action of RNAi; however, a partially modified pattern (25–75%) may increase the efficiency of RNAi and resistance to ribonuclease (Hall et al., 2004, 2006). At the same time, according to the work of Hall et al. (2004), where unpublished comparison data of PS and BP modifications of siRNA is mentioned, BP was shown to exhibit approximately twice as effective protection of siRNA from nucleases. If so, this modification could address some of the issues regarding the biomedical use of siRNA. One of the main limitations of the use of BP for siRNA modification is the lack of an optimized method of synthesizing large quantities of BP-modified siRNA; therefore, novel methods must be developed to assess the therapeutic potential of BP-modified siRNAs *in vivo*.

The introduction of modifications replacing the phosphodiester bond with an amide bond (Selvam et al., 2011) contributes to the protection of siRNA from the action of nucleases (Iwase et al., 2007), but their effect on the efficiency of RNAi is uncertain. The introduction of an amide bond between 10, 11, and 12 nucleotides, despite the absence of a phosphodiester bond, has been shown to increase the inhibitory effect of the modified siRNA, presumably due to the formation of additional hydrogen bonds between the amide group and Ago2 (Mutisya et al., 2017).

The presence of phosphate at the 5′ end of the “guide” strand of siRNA is essential for RNAi (Frank et al., 2010), while siRNA with 5′-hydroxyl possesses biological activity, since such siRNA is effectively phosphorylated inside cells (Weitzer and Martinez, 2007). When blocking phosphorylation of the 5′-hydroxyl, siRNA does not exhibit interfering activity (Chen et al., 2008). Chemical modifications of the first nucleotide from the 5′ end of the antisense strand can interfere with intracellular phosphorylation (Allerson et al., 2005; Chen et al., 2008; Kenski et al., 2012); however, the introduction of chemically stable phosphate [5′-(*S*)-C-methyl (Prakash et al., 2015), 5′-methylphosphonate (Lima et al., 2012), and 5′(*E*)-vinylphosphonate (Elkayam et al., 2017)] at the 5′ end of the antisense strand can restore activity (Lima et al., 2012; Prakash et al., 2015). The introduction of 5′(E)-vinylphosphonate solves this problem most efficiently. It has been shown that such modification of the antisense strand not only improves binding to Ago2 (Elkayam et al., 2017) 2-fold, but also leads to an increase in the accumulation and stability of siRNA conjugates containing cholesterol (Haraszti et al., 2017) or N-acetylgalactosamine (Elkayam et al., 2017) in the organs of mice following subcutaneous injection. Therefore, 5′(*E*)-vinylphosphonate modification of the antisense strand is a promising strategy for the development of therapeutic drugs based on siRNA.

### Nucleobase Modifications

Substitutions of nucleobases with various modified analogs [pseudouridine, 2′thiouridine, dihydrouridine (Sipa et al., 2007), 2,4-difluorobenzene (Somoza et al., 2006), 4-methylbenzimidazole (Somoza et al., 2008), hypoxanthine (Addepalli et al., 2010), 7-deazaguanin (Eberle et al., 2008), N^2^-alkyl-8-oxoguanine (Kannan and Burrows, 2011), N^2^-benzyl-guanine (Puthenveetil et al., 2006), and 2,6-diaminopurine (Chiu and Rana, 2003)] are designed to increase the thermal stability of the duplex by increasing the efficiency of the formation of hydrogen bonds with complementary nucleotides. However, such modifications reduce the efficiency of RNAi and do not contribute to an increase in siRNA resistance to nuclease action (Peacock et al., 2011). Nucleobase modifications in small amounts (up to 10%) could reduce immune reactions and improve the thermodynamic siRNA profile (Sipa et al., 2007; Anderson et al., 2010). The presence of such modifications in the patterns of chemical modification of siRNA can contribute to the optimization of the therapeutic properties of siRNA; however, this approach has not yet found wide application as similar effects can be obtained by introducing other modifications.

## Patterns of Chemical Modifications of siRNAs

siRNA is degraded *in vivo* as a result of its cleavage by endonucleases on pyrimidines (Turner et al., 2007) and exonucleases from both the 3′ and 5′ ends (Hsu and Stevens, 1993; Terrazas et al., 2013); thus, it is essential that siRNAs contain chemical modifications at cleavage sites to improve siRNA nuclease resistance to achieve biological activity of bioconjugates *in vivo*. However, the introduction of certain chemical modifications in siRNA is limited by inhibition of its interfering activity and toxicity. Thus, the introduction of modifications in siRNA is determined by the balance between the number of modifications sufficient for siRNA to be non-toxic, while retaining interfering activity and nuclease resistance. Introducing the 2′O-Me modification into siRNA can lead to inhibition of RNAi if the siRNA contains more than two consecutive 2′O-Me modifications in a row (Czauderna et al., 2003; Manoharan et al., 2011), while 2′O-Me modification of every second nucleotide does not block RNAi (Czauderna et al., 2003). Thus, an important parameter affecting RNAi is not only the number of introduced modifications, but also their location in the duplex.

One of the approaches to finding a balance between interfering activity and nuclease resistance of siRNA with 2′O-Me modifications is 2′O-Me selective modification of nuclease-sensitive siRNA sites (Volkov et al., 2009). siRNA is subjected to serum cleavage at pyrimidines (Turner et al., 2007); however, replacing all pyrimidines with 2′O-Me analogs completely inhibits the interfering ability of siRNA (Manoharan et al., 2011). It has been shown that the main sites of siRNA cleavage in serum are generally CA, UA, and UG sites. Introduction of 2′O-Me at these sites will preserve the interfering activity of siRNA, increase serum nuclease resistance (Volkov et al., 2009), and provide long-term suppression of target gene expression (Petrova Kruglova et al., 2010; Chernikov et al., 2017).

The main limitation of the introduction of 2′F modifications in siRNA is their toxicity, although siRNA conjugates containing 2′F modifications on pyrimidines are protected from the action of ribonucleases and, unlike 2′O-Me (Manoharan et al., 2011), possess biological activity (Wolfrum et al., 2007). The introduction of PS into siRNAs is also limited by the toxicity of the resulting oligonucleotides, and since PS-modified siRNAs have been shown to be highly protected against exoribonucleases (Eckstein, 2014; Kel'in et al., 2016), PS modification is used only to replace two or three terminal nucleotides in siRNA (Soutschek et al., 2004). Most of the other chemical siRNA modifications are primarily introduced along the terminal regions of the duplex for various reasons; e.g., the effect of RNAi on proteins, thermal asymmetry, and protection against nucleases (Deleavey and Damha, 2012). It is important that modifications designed to change the properties of siRNAs that are significant for its therapeutic potential can be used together, complementing one another and ensuring biological activity and siRNA resistance to nucleases more efficiently than each modification separately (Deleavey et al., 2010). Recent studies have used combinations of chemical modifications to achieve maximum effect *in vivo*.

Since after replacing each second nucleotide with the 2′O-Me analog, half of the siRNA molecule is unprotected from the action of nucleases, the introduction of 2′F modifications into the unmodified portion of the duplex was proposed. It has been shown that siRNA molecules with alternating 2′O-Me and 2′F modifications are stable in mouse plasma and suppress expression of the target gene by several orders of magnitude more efficiently compared to unmodified siRNA (Allerson et al., 2005). Successful use of fully modified siRNA molecules based on alternating 2′O-Me and 2′F modifications was demonstrated when studying the properties of single-stranded siRNAs (ssRNAs). ssRNAs with this pattern, with the addition of several PS, 2′O-MOE, and 5′ (*E*)-vinylphosphonate modifications, exhibited biological activity *in vitro* and *in vivo* (Lima et al., 2012; Prakash et al., 2015), and modeling of the complex of this siRNA with Ago2 showed that the modifications did not sterically block the interaction of the ssRNA with Ago2 (Schirle et al., 2016).

**Figure 3:**
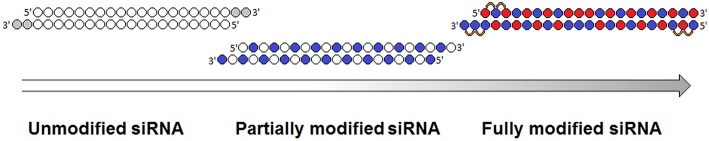
Evolution of siRNA chemical modification patterns. Chemical modifications are indicated in gray (deoxyribonucleotides), blue (2′O-Me), red (2′F), and orange (PS) [adapted from Khvorova and Watts (2017)].

Despite the fact that duplex cleavage in serum at internal nuclease sensitive sites makes the greatest contribution to siRNA degradation, siRNA cleavage can also occur at other sites. A fully modified siRNA, containing alternating 2′O-Me and 2′F modifications, was compared with a partially modified siRNA, containing a combination of 2′O-Me and 2′F modifications located along the pyrimidines and the ends of the duplex; both patterns contained PS modifications on the 3′-overhangs (Hassler et al., 2018) (Figure 3). A cholesterol conjugate of the fully modified siRNA more effectively reduced expression of the *Htt* gene in HeLa cells compared to a cholesterol conjugate of the partially modified siRNA; the concentrations of siRNA at which expression of the target gene was 50% suppressed were 70 and 170 nmol/l, respectively (Hassler et al., 2018).

In contrast to serum siRNA cleavage, siRNA degradation in lysosomal hepatocyte extracts occurs mainly by 5′-exonucleases; therefore, siRNA could be further stabilized by PS modifications of the 5′ ends in the N-acetylgalactosamine conjugate to increase the duration and efficiency of its inhibitory effect (Nair et al., 2017). Subcutaneous administration of 10 mg/kg of conjugate of fully modified at 2′ ribose positions siRNA and N-acetylgalactosamine was shown to cause a 30% decrease in target gene expression at the protein level for 10 days, while addition of the conjugate with modified 5′ ends reduced the protein level by 80% for 40 days. Conjugates of fully modified at the 2′ positions and stabilized by PS modifications at both the 3′ and 5′ ends siRNAs with cholesterol and docosahexaenoic acid were examined *in vivo* (Hassler et al., 2018). The accumulation of conjugates in the liver, kidney, and spleen 24 h after intravenous and subcutaneous injections was studied. Accumulation of all mentioned above conjugates was two orders of magnitude higher compared to similar conjugates of partially modified siRNA (2′O-Me and 2′F modifications located on pyrimidines and duplex ends and PS modifications on 3′-overhangs). However, no control experiment was performed to determine the contribution of the introduction of PS at the 5′ ends to the stability of siRNA conjugates in this study. Since the contribution of 2′O-Me modifications to the nuclease resistance of siRNA is greater than that of 2′F modifications (Cummins et al., 1995; Takahashi et al., 2009), attempts were made to increase the proportion of 2′O-Me modifications in siRNAs containing 50% 2′O-Me and 50% 2′F (Khvorova, 2017; Foster et al., 2018). For this purpose, 1,890 different siRNAs were synthesized, aimed at five different genes, varying in sequence (15 variants) and pattern of 2′O-Me and 2′F modifications (Foster et al., 2018). The optimal introduction of 2′O-Me or 2′F modifications for each position in the siRNA was determined via *in vitro* analysis of primary mouse hepatocytes. Two modification patterns were selected, containing 23% and 18% 2′F modifications, the biological activities of which were not less than that of the parent siRNA. Analysis of the biological activities of three different siRNA sequences *in vivo* showed that a lower content of 2′F modifications (18%) was the most effective. Subcutaneous administration of 1 mg/kg of the siRNA and N-acetylgalactosamine conjugate to primates showed that the newly selected siRNA (18% 2′F modifications) reduced the protein level by ~70% for 70 days, while the parent siRNA suppressed the expression of the target gene *AT* by ~40% for 40 days. It has been shown that a conjugate of fully modified siRNA (23% 2′F, 73% 2′O-Me, and one dNMP) and N-acetylgalactosamine suppressed *PCSK9* gene expression in the liver of patients following a single subcutaneous injection of ~6 mg/kg by ~70% for 6 months (Ray et al., 2017).

When siRNAs are delivered as part of a bioconjugate, they are especially sensitive to the action of nucleases, and the duration of biological action *in vivo*, and the dose and frequency of drug administration depends on nuclease resistance. Therefore, it is important to pay particular attention to this parameter when creating therapeutic drugs based on siRNA bioconjugates.

## Bioconjugates

**Figure 4:**
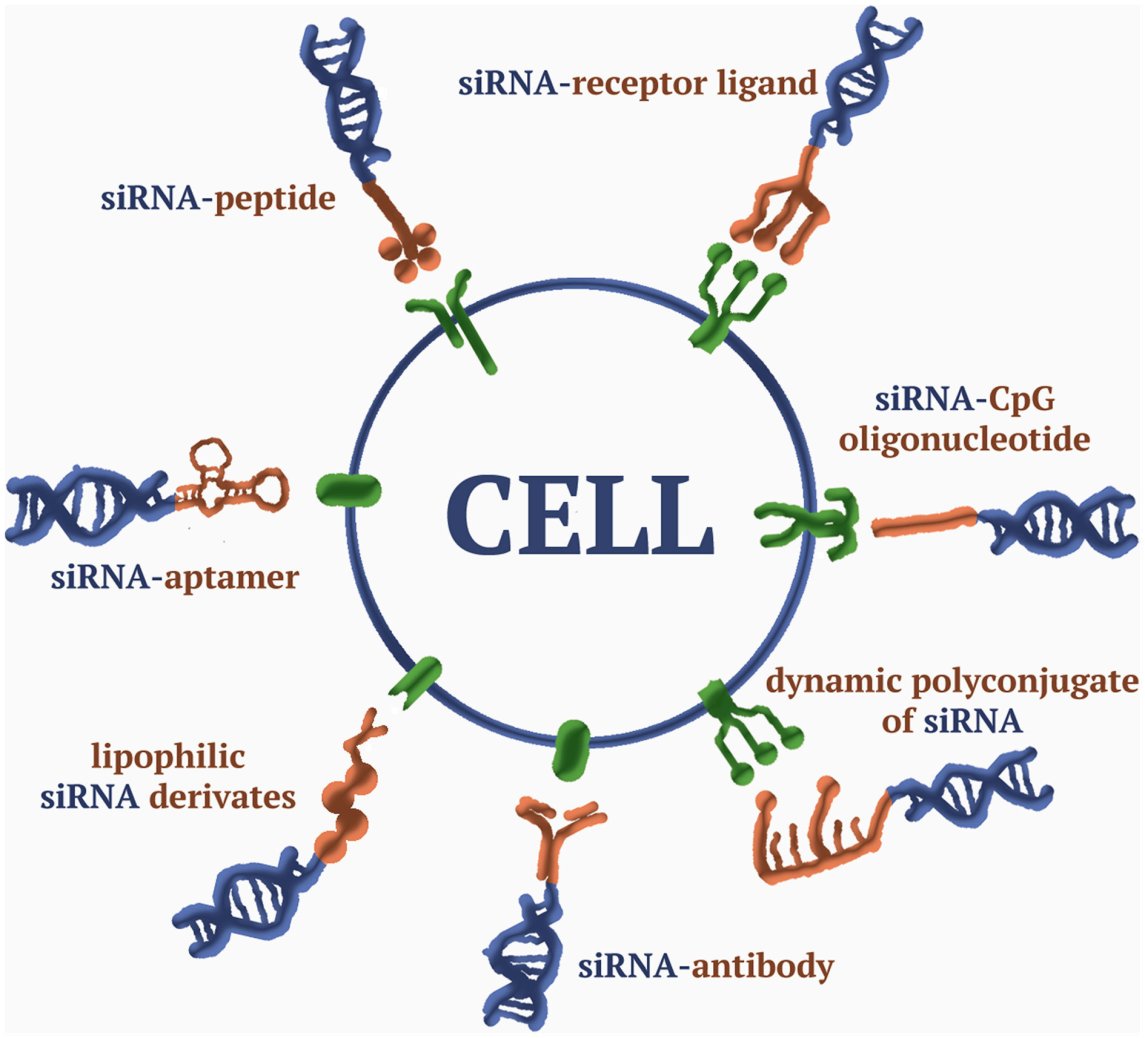
A scheme of siRNA bioconjugates.

The use of bioconjugation as a method of delivering siRNA to cells involves forming siRNA conjugates with (1) biomolecules capable of specifically binding receptors on the cell membrane [folate Thomas et al., 2009, antibodies Song et al., 2005; Dassie et al., 2009; Xia et al., 2009, aptamers Aronin, 2006; McNamara et al., 2006, some peptides Cesarone et al., 2007; Lau et al., 2012, and carbohydrates Nair et al., 2014], (2) molecules able to penetrate the cell by natural transport mechanisms [cholesterol (Lorenz et al., 2004) and vitamins Nishina et al., 2008], or (3) molecules capable of interacting non-specifically with the cell membrane [positive electrostatic charge and hydrophobicity Kwiatkowska et al., 2013; Meade et al., 2014] (Supplementary Table 1; Figure 4). In addition to the nature of the biogenic molecule, the structure of the linker that binds the siRNA and the biomolecule affects the efficiency of accumulation and the biological activity of the siRNA. In particular, the ability of the linker to be cleaved when the conjugate enters the cells prevents a decrease in the efficiency of RNAi associated with the inhibition of RISC assembly. Disulfide (Turner et al., 2005) and thioether bonds (Meade et al., 2014), pH sensitive bonds [hydrazone (Dovydenko et al., 2016), carboxymethylmaleic anhydride Rozema et al., 2007], or photosensitive bonds (β-[bis (4-methoxyphenyl)-phenylmethoxy]-2-nitrobenzeneethanol linker Yang et al., 2018) are used as cleavable bonds. Conjugates containing linkers that are stable under the experimental conditions (Lorenz et al., 2004) are widely used, and the structure of the conjugate plays a key role. The most commonly used types of bioconjugates are reviewed below.

### Lipophilic siRNA Derivatives

Lipids and cholesterol were suggested as the first ligands for conjugation with siRNAs, since they were supposed to ensure the interaction of siRNAs with the cell membrane due to their lipophilic properties and because of endogenous transport mechanisms (Letsinger et al., 1989). Cholesterol is not only part of the membrane, but is also transported into cells by low-density lipoproteins (LDL particles) and high-density lipoproteins (HDL particles) (Brunzell et al., 2008), which bind to corresponding receptors. Absorption of all lipoproteins by cells is carried out through the recognition of protein components of the particles by clathrin-dependent receptor-mediated endocytosis, using LDL and scavenger receptor class B member 1 (SR-BI) receptors that recognize LDL and HDL particles, respectively (Goldstein et al., 1985; Yvan-Charvet et al., 2008).

It has been shown that siRNA and cholesterol derivatives, as well as other lipophilic siRNA derivatives, are able to form complexes with HDL and LDL particles under certain conditions, which, in turn, can bind to the corresponding receptors (Wolfrum et al., 2007). It has also been shown that the hydrophobicity of a lipophilic molecule determines the efficiency of binding a lipophilic conjugate with lipoproteins (Wolfrum et al., 2007). Thus, a cholesterol residue introduced into siRNA provides effective binding to LDL and HDL particles and, as a result, higher activity compared to other lipophilic derivatives. However, the assumption of the penetration of lipophilic siRNA derivatives in the composition of such complexes into cells by receptor-mediated endocytosis has not been confirmed (Wolfrum et al., 2007). The transmembrane protein SIDT1 (Wolfrum et al., 2007) participates in the penetration of complex of lipophilic siRNA derivatives and lipoproteins. SIDT1 facilitates the penetration of dsRNA into cells, by forming channels for diffusion or by facilitating the penetration of dsRNA indirectly via interaction with other proteins (Feinberg and Hunter, 2003; Wolfrum et al., 2007). On the other hand, SIDT1 has a binding domain that interacts with steroid molecules and its localization in the cell depends on the presence or absence of cholesterol in the membrane (Mendez-Acevedo et al., 2017). It has also been shown that the SIDT1 homolog SIDT2 is involved in cellular transport of cholesterol (Mendez-Acevedo et al., 2017) and dsRNA without lipids (Nguyen et al., 2017; Takahashi et al., 2017). However, the specific role of SIDT1 in the penetration of complexes of lipophilic siRNA derivatives with lipoproteins has not been established.

Penetration of siRNA cholesterol conjugates without a carrier was studied in HeLa cells (Gilleron et al., 2015). For this purpose, the expression of genes important for endocytosis (*DNM1L, CLTC, CAV1, CDC42*, and *RAC1*) in cells was suppressed, and accumulation of the fluorescently labeled cholesterol conjugate was evaluated. Accumulation of the cholesterol conjugate was reduced by ~40% compared to accumulation in untreated cells only when expression of *DNM1L* and *CLTC*, which participate in clathrin-dependent endocytosis, was inhibited.

In another study (Ly et al., 2017), clathrin-dependent intracellular transport of cholesterol conjugates was investigated by determining the colocalization of fluorescently-labeled endosome proteins and ligands of clathrin-dependent endocytosis with a fluorescently-labeled cholesterol conjugate. In this work, clathrin-dependent endocytosis was shown to account for 25% of the total intracellular transport of the cholesterol siRNA derivative in the cell. It should be noted that endocytosis is characterized by the ability to sort absorbed endosomal contents for recirculation or degradation (Lakadamyali et al., 2006). Depending on the type of receptor and the content of the endosome, sorting can occur at different stages of endocytosis; sorting and recycling of the LDL receptor takes an average of ~6 min (Brown and Goldstein, 1976). Analysis of the kinetics of accumulation of cholesterol-modified siRNA revealed that in the first 60 min after the addition of cholesterol derivatives of siRNA to HeLa cells, only 5% of the conjugate was recycled, and 20% of the conjugate was sorted to the degradation pathway (Ly et al., 2017). Thus, it can be assumed that with systemic administration of the cholesterol conjugate, the primary route of transport is interaction with lipoproteins and penetration by receptor-mediated endocytosis into cells expressing the corresponding receptor (LDL or SR-BI receptor). Then, likely at some stage of intracellular transport, lipoproteins and the cholesterol-siRNA conjugate dissociate and the siRNA enters the cytoplasm, where it participates in RNAi. Indeed, many studies have shown that the addition of cholesterol to the 5′ and 3′ ends of the sense strand and the 3′ end of the antisense strand provides manifestation of the biological activity of siRNA upon delivery without a carrier *in vitro* (Lorenz et al., 2004; Cesarone et al., 2007; Moschos et al., 2007; Alterman et al., 2015; Chernikov et al., 2018) and *in vivo* (Soutschek et al., 2004; Wolfrum et al., 2007; Byrne et al., 2013; Khan et al., 2016; Haraszti et al., 2017). In most cell lines, the cholesterol-siRNA conjugate shows higher biological activity compared with other conjugates of siRNA and lipophilic derivatives; e.g., lithocholic acid derivatives, saturated fatty acids (C12–C22) (Wolfrum et al., 2007; Prakash et al., 2015), unsaturated fatty acids (Nikan et al., 2016, 2017), or tocopherol (Nishina et al., 2008).

The biodistribution of various lipophilic siRNA conjugates has been extensively studied in a recent paper (Biscans et al., 2018). It was shown that cholesterol conjugates were more effectively retained in the body (62%) compared with other lipophilic conjugates (27–62%). Following subcutaneous injection, the cholesterol conjugates accumulated in almost all organs: liver, kidney, adrenal glands, spleen, pancreas, heart, muscle, fat, thymus, lung, injection site, ovaries, and testes. At the same time, cholesterol conjugates accumulated most effectively in the liver, adrenal glands, spleen, and in the skin at the site of administration (Biscans et al., 2018). Cholesterol derivatives accumulated in other organs with the same or lesser efficiency than other lipophilic conjugates; e.g., it was shown that a conjugate of siRNA and saturated fatty acid (docosanoic, C21) accumulated more efficiently than a cholesterol conjugate and inhibited expression of the target gene (*Htt* or *Ppib*) in muscles (20 and 30% inhibition of *Htt* and *Ppib*, respectively) and fat (50% and 30% inhibition of *Htt* and *Ppib*, respectively) after subcutaneous injection (20 mg/kg) (Biscans et al., 2018). In this study, it was shown that the main factor determining the nature of the biodistribution of conjugates is their lipophilicity. Conjugates of siRNA with lower lipophilicity; i.e., derivatives of retinoic acid, lithocholic acid, and docosahexanoic acid with greater efficiency than cholesterol conjugates accumulated in the kidneys, bladder, and lungs of the mouse after subcutaneous injection (Biscans et al., 2018). This fact is consistent with previous data that showed that more lipophilic conjugates bind more efficiently to serum components, and thus are not excreted by the kidneys (Wolfrum et al., 2007; Osborn et al., 2018).

Lipophilic derivatives after subcutaneous or intravenous injection do not penetrate the blood-brain barrier (BBB) (Biscans et al., 2018). Therefore, attempts were made to directly inject derivatives into the brain of the mouse to suppress gene expression in the brain (Alterman et al., 2015; Nikan et al., 2016, 2017). Since docosahexaenoic acid is the most common polyunsaturated fatty acid in the mammalian brain, conjugation of siRNA with docosahexaenoic acid more effectively suppressed the expression of the target gene (Nikan et al., 2016) than other lipophilic conjugates (Alterman et al., 2015). Injection of the siRNA-docosahexaenoic acid conjugate into the brain striatum of the mouse (~1.25 mg/kg) caused a decrease in the mRNA level of the target gene (*Htt*) not only in the striatum (73%) but also in the cortex (52%) of the corresponding hemisphere (Nikan et al., 2016). However, no decrease in *Htt* mRNA was observed in the striatum and the cortex of the opposite hemisphere. An assessment of the toxicity of the conjugate in the brains of animals, at a dose 20 times higher than that in a study of biological activity, showed that this conjugate does not elicit an immune response or neuronal death. The therapeutic significance of suppressing the expression of the *Htt* gene was shown in another study in a mouse model of Huntington's disease using ASO (Kordasiewicz et al., 2012). It was shown that suppression of the expression of both alleles (mutant and wild-type) resulted in restoration of a healthy animal phenotype. Therefore, the use of docosahexaenoic acid for conjugation with siRNA may be a promising approach for the treatment of various hereditary neurodegenerative diseases, including Huntington's disease. Thus, such conjugates can be considered as a universal platform for siRNA delivery throughout the body since lipophilic siRNA derivatives can accumulate and exhibit biological activity in a variety of tissues and organs.

### siRNA and Peptide Conjugates

Some proteins and peptides are able to penetrate into the cell due to endogenous transport mechanisms, as well-transfer other molecules into the cell. The main mechanisms of peptide transport include binding to surface proteins, glycoconjugates [targeted peptides Pooga et al., 1998; Alberici et al., 2013], or anionic cell lipids, followed by absorption by endocytosis, membrane penetration (cell penetrating peptides [CPPs] Vives et al., 1997; Thoren et al., 2000; Console et al., 2003; Heitz et al., 2009; van den Berg and Dowdy, 2011; Lee et al., 2013; Gagat et al., 2017), membrane lysing, or pore formation in the membrane [lytic peptides (Meyer et al., 2009)]. There are two main ways of obtaining such peptides: using phage display, or using parts of proteins that perform similar functions in nature. In this section, the main approaches for siRNA delivery by peptide are reviews.

The ability of peptides to specifically interact with certain proteins on the cell surface due to specific elements in their tertiary structure was used for targeted delivery of siRNAs. Different targeted peptides were conjugated to siRNAs and such conjugates possessed biological activity *in vitro* (Cesarone et al., 2007; Alam et al., 2011; Alberici et al., 2013) and *in vivo* (Liu et al., 2014). For example, a conjugate of siRNA and the peptide “CSKC,” which mimics insulin-like growth factor 1 (IGF-1), effectively penetrated MCF7 cells expressing the IGF-1-specific receptor and suppressed expression of the *IRS1* target gene by 60% without the help of transfection agents (Cesarone et al., 2007).

One of the most successful examples of targeted peptides is the cyclic RGD (cRGD) peptide. cRGD is part of the iRGD peptide obtained by selecting a phage library of cyclic peptides for binding to a xenograft PC-3 tumor (Sugahara et al., 2009). cRGD binds to αVβ3/5 integrins that are expressed at a high level in tumor cells and vascular endothelium cells (Dubey et al., 2004; Weis and Cheresh, 2011). Conjugation of this peptide with siRNA contributed to the accumulation and manifestation of the biological activity of siRNA in tumor cells in culture (Alam et al., 2011) and *in vivo* following six intravenous injections in animals with xenograft A549 tumors (reduction of *VEGFR2* gene mRNA by 55%), which was accompanied by a decrease in tumor growth (Liu et al., 2014) (Supplementary Table 1). Attempts to increase the number of RGD peptides in the siRNA conjugate were made; however, the introduction of additional molecules of this peptide did not have a direct dose-dependent effect on biological activity. Assessment of biological activity to suppress expression of the *GFP* gene in M21^+^GL_3_ cells showed that a siRNA conjugate with two cRGD peptides has negligible activity (20%), a conjugate with four cRGD peptides showed moderate biological activity (37%), and a conjugate with three cRGD peptides had the highest biological activity (73%) (Alam et al., 2011).

Covalent attachment of peptides can not only increase the efficiency of siRNA accumulation in cells, but also ensure the specificity of their actions in target cells. A peptide with the “LEVDG” sequence attached to siRNA blocks RISC (Koehn et al., 2010) assembly; however, this sequence is specifically recognized and cleaved by caspase-4, which is expressed in Jeg-3 choriocarcinoma cells. Thus, following the introduction of anti-*STAT3* siRNA conjugated with the “LEVDG” peptide into Jeg-3 cells, effective (up to 70%) suppression of target gene expression was observed (Supplementary Table 1), whereas in the control HEK293 cells not expressing caspase-4, suppression of the *STAT3* gene was not observed (Koehn et al., 2010).

Due to the presence of positively charged amino acids in its composition and the secondary structure, CPP peptides, such as penetratin (Moschos et al., 2007), transportan (Muratovska and Eccles, 2004) and trans activator of transcription (Tat) (van den Berg and Dowdy, 2011) are able to penetrate the cell membrane, as well as deliver covalently attached nucleic acids into cells (Chiu et al., 2004; Muratovska and Eccles, 2004). It has been shown in a number of studies (Muratovska and Eccles, 2004; Cesarone et al., 2007) that CPP-siRNA conjugates exhibit biological activity when added to cells. However, the use of such conjugates *in vivo* is limited because they are toxic and can elicit an immune response (Boeckle et al., 2005; El-Andaloussi et al., 2007; Moschos et al., 2007).

Another limitation to the use of CPPs as siRNA delivery agents is the formation of complexes of positively charged CPP with siRNA, which prevents the siRNA from interacting with RISC components. To avoid this, neutralizing or shielding the negative charge of the siRNA by introducing chemical modifications [*tert*-butyl-S-acyl-2-thioethyl phosphotriester (*t*Bu-SATE) (Table 1, 2)] has been proposed (Meade et al., 2014; Hamil and Dowdy, 2016). The introduction of *t*Bu-SATE modifications into the siRNA conjugate made it possible to obtain siRNA conjugates with several cationic peptides without inhibiting RNAi. At the same time, the biological activity of such conjugates directly depended on the number of CPP molecules of the Tat peptide present (Meade et al., 2014). Conjugates containing four Tat peptides more effectively suppressed the expression of a target gene in cells compared with a conjugate with two or three Tat peptides. However, despite promising *in vitro* results (Meade et al., 2014; Kolosenko et al., 2017), this siRNA conjugate has not yet been studied *in vivo*.

Another successful example of the use of CPP as a ligand for conjugation with siRNA is the skin penetrating and cell entering (SPACE) peptide, obtained by the phage display method by selecting peptides that penetrate the epidermis (Hsu and Mitragotri, 2011). Attachment of the SPACE peptide to siRNA promoted penetration of the siRNA through the epidermis and dermis following application on the skin surface. A single application of ~12 mg/kg of the siRNA conjugate with the SPACE peptide suppressed the expression of *IL10* and *GAPDH* in the epidermis by 28 and 47%, respectively. The inclusion of such a conjugate in the composition of lipoplexes enriched with the SPACE peptide results in a more effective downregulation of the expression of the target gene (*GAPDH*).

To solve the problem of effective *in vivo* accumulation of siRNA, an interesting approach using siRNA containing a chemical modification at the 3′ end of the sense strand capable of forming a covalent bond between siRNA and albumin following intravenous injection of siRNA was proposed (Lau et al., 2012). For this purpose, succinimidyl 4-[N-maleimidomethyl] cyclohexane-1-carboxylic acid was attached to siRNA using the amino group at the 3′ end of the sense strand; the resulting molecule (“activated siRNA”) could interact with albumin to form a disulfide bond. When BALB/c mice were injected with the “activated siRNA” capable of binding albumin, there was a more efficient accumulation of siRNA in the myocardium compared to unmodified siRNA, as well as a decrease in the mRNA level of the *IGF-IR* target gene by 35% (Supplementary Table 1). However, the toxic effect of the “activated siRNA” has not yet been investigated.

Another approach for the delivery of siRNA to target cells uses its conjugation with lytic peptides, which facilitate the release of siRNA from the endosome (Varkouhi et al., 2011). The main mechanisms that increase the efficiency of the release of siRNA from the endosome are (1) formation of transmembrane pores by peptides due to their amphiphilicity and ability to form complexes [melitin, cytolytic peptide from bee venom (Meyer et al., 2009), ricin, and ribosome-inactivating protein from the oil (Sun et al., 2004)]; (2) protonation of the main groups of peptides with a decrease in the pH of the endosome, followed by an increase in the osmotic pressure inside and rupture of its membrane [polyhistidine (Chen et al., 2017)]; or (3) local membrane destabilization due to the fusogenic properties of proteins and membrane penetration of the endosome [glycoprotein H from the herpes virus (Tu and Kim, 2008), hemagglutinin-2 domain of the influenza virus (Wadia et al., 2004; Lee et al., 2011), and diphtheria toxin domain (Barati et al., 2002)]. Despite the efficiency of the action of lytic peptides, the primary issue is toxicity, since the formation of pores and increased osmotic pressure inside the endosome implies its destruction. In this case, the approach in which the membrane is locally destabilized is less toxic, since it does not lead to significant damage to the endosome and therefore is the most promising. However, to date, an effective and non-toxic endosomolytic agent based on peptides has not been developed.

The use of siRNA and peptide conjugates for siRNA delivery is a promising approach; however, at present, the efficiency and specificity of delivery provided by peptides does not reach the level at which they do not exhibit toxic and immunogenic effects.

### siRNA and Receptor Ligand Conjugates

The main factors affecting the efficiency of specific delivery to target cells are the efficiency of ligand binding to the receptor and the degree of expression of the receptor on the membrane surface. Typically, the interaction of the ligand with the receptor is characterized by high specificity, so introduction of these molecules contributes to effective targeted delivery when covalently attached to molecules (Nikam and Gore, 2018). The most successful example of this strategy is the use of N-acetylgalactosamine as a ligand for siRNA delivery because its interaction with the asialoglycoprotein receptor (ASGPR) occurs with high efficiency (K_d_ = 2.5 nM) and this receptor is expressed at a high level in hepatocytes (0.5–1 × 10^6^ molecules per cell) (Spiess, 1990). Conjugation of siRNA with N-acetylgalactosamine contributes to efficient delivery of siRNA and the conjugate suppresses expression of the target gene (*PCSK9*) by 70% following a single subcutaneous injection of ~6 mg/kg (Ray et al., 2017) (Supplementary Table 1). The level of ASGPR in hepatocytes is so high that reducing its expression by 50% does not reduce the biological activity of the siRNA and N-acetylgalactosamine conjugate; only suppression by 95% blocks the action of this conjugate (Willoughby et al., 2018). For these reasons, and due to inexpensive synthesis, siRNA and N-acetylgalactosamine conjugates are among the most promising prototypic drugs for introduction in the clinic for the treatment of liver diseases.

Conjugation with folic acid was proposed for specific accumulation of siRNA in tumor cells (Thomas et al., 2009). Folic acid is a precursor of tetrahydrofolate, which is essential for the synthesis of nucleotides *de novo*, and thus is essential for dividing cells. Folic acid penetration into cells occurs via receptor-mediated endocytosis, via the glycoprotein folic acid receptor, which binds strongly to folate (K_d_ = 10^−10^ M). It has been shown that expression of folic acid receptor in tumor cells of different origin is significantly higher compared with the expression level in normal cells (Parker et al., 2005; Xia and Low, 2010). Presumably, the penetration of folate-containing siRNAs also occurs via receptor-mediated endocytosis (Low et al., 2008); therefore, the penetration efficiency of folate-containing siRNAs into tumor cells is significantly higher compared with the efficiency of conjugate penetration into normal cells. *In vivo*, fluorescently-labeled siRNA and folate conjugates were shown to accumulate more efficiently in a mouse tumor compared to unmodified siRNA (Thomas et al., 2009). However, the use of siRNA and folate conjugates is limited to experimental purposes due to sophisticated synthesis.

### siRNA and Aptamer Conjugates

Aptamers are synthetic oligoribonucleotides (molecular weight, ~6–30 kDa) with a complex tertiary structure, which allows specific binding to molecules (Zhou and Rossi, 2017). It has been shown that the attachment of aptamers to siRNAs contributes to specific accumulation in certain cell types (Catuogno et al., 2018). For instance, siRNA conjugation with the A10 aptamer specific to prostate specific membrane antigen (PSMA) promoted effective delivery of siRNA to prostate tumor cells, and the biological activity of the conjugate was comparable to that observed in the case of siRNA delivered by lipoplexes (McNamara et al., 2006). The conjugate silenced *PKL1* and *Bcl2* genes with 85 and 90% efficiency, respectively, in the xenographic prostate LNCaP tumor-bearing mice model after 10 intratumoral injections; a decrease in tumor growth and regression were also observed (McNamara et al., 2006) (Supplementary Table 1). In the same xenograft tumor model, another shRNA (short hairpin RNA) and aptamer A10-3 to PSMA conjugate also exhibited biological activity (65% inhibition of the *PRKDC* gene), but tumor regression occurred after two intratumoral injections only in combination with ionizing radiation (Ni et al., 2011) (Supplementary Table 1).

A conjugate of siRNA and the A-1 aptamer specific for the gp120 surface protein of human immunodeficiency virus 1 (HIV-1) capsid was synthesized as an anti-HIV-1 drug (Neff et al., 2011). In humanized Rag2^−/−^ γc^−/−^ (RAG-hu) mice 3 weeks after infection with HIV-1, weekly administration (0.38 mg/kg, intravenous) of this conjugate reduced the concentration of viral RNA in the plasma of animals by 105 times. However, a few weeks after treatment, the amount of viral RNA in the plasma was restored almost to the initial level (Supplementary Table 1).

Another interesting property of some aptamers is their ability to penetrate the BBB. For instance, it has been shown that the aptamers Gint4.T and GL21.T, specific to beta-type platelet-derived growth factor receptor (PDGFRβ), can penetrate the *in vitro* model of the BBB (Esposito et al., 2016). This siRNA conjugate is able to accumulate in xenograft glioblastomas after several intravenous injections, suppress expression of the target gene (*STAT3*) by 60%, and reduce the rate of tumor growth (Esposito et al., 2018).

In addition to RNA aptamers, DNA aptamers have also been used for the delivery of siRNAs (Lai et al., 2014). The G-quadruplex-forming G-rich deoxyoligonucleotide AS1411 specifically binds nucleolin, an oncogene protein expressed at a high level in many cancer cell types (Ireson and Kelland, 2006). The AS1411-based DNA aptamer aptNCL conjugated with siRNA enables its delivery and biological activity in lung cancer cells *in vitro* and *in vivo* (Lai et al., 2014).

Although siRNA and aptamer conjugates have high biological efficiency at the experimental level, their use in the clinic has been limited by such factors as nuclease cleavage, filtration by the kidneys, polyanion effects, and the immune response. Selection of a new specific sequence of an aptamer to a specific object (systematic evolution of ligands by exponential enrichment [SELEX]) is fast, but the resulting aptamers do not always have high specificity for the target antigen (Yan and Levy, 2018). Nevertheless, in most cases, conjugation of siRNA with aptamers provides reproducible specific delivery of siRNA to target cells, and the possibility of obtaining aptamers directed to any protein on the surface of the cell membrane suggests this may be a promising approach. Therefore, the use of a modified SELEX protocol to search for chemically modified aptamers and conjugation with fully modified siRNAs can increase the effectiveness and duration of the therapeutic effect of aptamer-based conjugates of siRNA and their introduction into clinical practice (Hori et al., 2018).

### Antibody-siRNA Conjugates

Antibody-siRNA conjugates (ARCs) have been successfully used for targeted delivery of siRNA to specific types of cells expressing receptor-antigens; however, the effectiveness of ARCs varies significantly. For example, it has been shown that an ARC with an antibody against the insulin receptor suppresses expression of the target gene by 90% in HEK293 cells at a concentration of 115 nM (Supplementary Table 1) (Xia et al., 2009). Another ARC with an antibody to the Lewis-Y protein inhibited the expression of the target gene by 60% at a concentration of 300 nM only when the cells were treated with chloroquine, an agent that inhibits endosome maturation (Supplementary Table 1) (Ma et al., 2011). However, the non-covalent siRNA complex with the same antibody, formed by electrostatic interaction of oligo-arginine and siRNA, showed 60% biological activity (300 nM) in the absence of chloroquine. It is likely that the efficiency of endosomal escape mediated by chloroquine or oligo-arginine is an important factor for the biological activity of ARCs. The biological activity of both covalent and non-covalent siRNA complexes and antibodies has been shown *in vivo* in a number of studies (Song et al., 2005; Xia et al., 2007; Baumer et al., 2015; Sugo et al., 2016; Ibtehaj and Huda, 2017). However, a systemic comparison of the effectiveness of the biological activity of ARCs differing in target antigens was carried out only in one study (Cuellar et al., 2014), which showed that along with the level of expression of the receptor-antigen, the type of intracellular transport of the receptor influences the interfering activity of the ARC. However, no direct correlation was found between the type of penetration of the antibody complex with the receptor-antigen and the biological activity of the ARC. Such a factor as the presence of a cleavable bond between the siRNA and antibody did not affect the interfering activity of the ARC. Since in this study (Cuellar et al., 2014), the efficiency of the binding of antibodies to the corresponding receptors was not compared, it is not possible to evaluate the efficiency of their dissociation and the degree of endosomal escape of the conjugates. However, it is likely that this is a significant factor in determining the biological activity of ARCs.

The mechanism of penetration into cells of an ARC with the TENB2 antibody exhibiting high biological activity was studied (Cuellar et al., 2014). It was shown that the silencing of genes associated with clathrin-dependent receptor-mediated endocytosis led to a decrease in the efficiency of gene silencing by ARC. However, the suppression of the expression of *RAB5C* and *HPS4*, which are associated with intracellular transport, increased the silencing activity of the ARC. The product of the *RAB5C* gene likely sorts endosomal contents to the recycling pathway (Chen et al., 2014), while the product of the *HPS4* gene is involved in regulation of RNAi (Lee et al., 2009). It has been shown that the product of the *HPS4* gene reduces the amount of RLC and RISC proteins in the cell by increasing the frequency of lysosomes merging with multivesicular bodies, where, presumably, RNAi proteins are located (Lee et al., 2009). Thus, the suppression of *HPS4* gene expression leads to an increase in the efficiency of RNAi in cells and a corresponding increase in the activity of the conjugate. The effectiveness of *PPID* gene silencing by the ARC with the TENB2 antibody after three intravenous injections of 24 mg/kg in xenograft PC3-TENB2-high tumor-bearing nude mice was only 33% (Supplementary Table 1) (Cuellar et al., 2014).

In another study (Sugo et al., 2016), an F_ab_ antibody fragment, an immunoglobulin molecule segment that binds an antigen that has lower affinity for the target receptor than antibody, was used for conjugation with siRNA. After 4 weekly intramuscular injections (~3.6 mg/kg) of the conjugate of siRNA and F_ab_ fragment to the transferrin receptor, the *MSTN* mRNA level was decreased by 72%, which increased the average running distance of mice by 24% in the peripheral arterial disease model (Sugo et al., 2016). Intramuscular injection of only ~0.05 mg/kg of the conjugate of siRNA and F_ab_ fragment to the transferrin receptor resulted in suppression of the *HPRT* target gene at the injection site by 55%. High biological activity of the siRNA and F_ab_ fragment conjugate in muscle cells following intravenous injection was demonstrated by Avidity Bioscience. The mRNA level of the target *MSTN* gene was decreased by 90% and lasted for 20 days following a single intravenous injection of this conjugate, while the antigen for the F_ab_ fragment was also a transferrin receptor. F_ab_ fragments more efficiently than antibodies escape endosomes into the cytoplasmic space after being absorbed by cells. This is likely due to lower receptor binding efficiency and low molecular weight (55 kDa). Therefore, this approach is promising for the targeted delivery of siRNA to cells; however, a direct comparison of ARCs with conjugates of siRNA and F_ab_ fragments has not yet been carried out.

Conjugation of siRNAs with antibodies to deliver siRNA to target cells has several advantages compared with the conjugation of siRNAs with other molecules, such as high ligand binding efficiency (K_d_ < 10^−9−10^) and prolonged presence in blood due to high molecular weight (~150 kDa). However, the immune response and low efficiency of the endosomal yield are the main disadvantage of this approach. Thus, further optimization, including the use of humanized antibodies or F_ab_ fragments, endosomolytic agents, and fully modified siRNAs, is required for effective use of ARCs in the clinic.

### siRNA and CpG Oligonucleotide Conjugates

As an alternative method of siRNA delivery to target cells, systems that provide an efficient release of siRNA from endosomes to the cytoplasm are used. For example, conjugation of DsiRNA with CpG-containing oligodeoxyribonucleotides leads to recovery of the interfering activity of DsiRNA in cells expressing the TLR9 receptor due to endosomal release mediated by TLR9 (Nechaev et al., 2013). Thus, the conjugate is biologically active only in cells expressing the TLR9 receptor, such as cells of the immune system: B-lymphocytes, dendritic cells, and macrophages, as well as in some types of cancer (Zhang et al., 2013). The therapeutic effect of the siRNA and CpG oligonucleotide conjugate has been shown in various tumor-bearing mouse models following systemic administration of the conjugate over several weeks (Kortylewski et al., 2009; Zhang et al., 2013; Hossain et al., 2014). However, since the injection of CpG oligonucleotides leads to the activation of cytokines and interleukins, their use is limited. Also, its application *in vivo* is limited to local injections due to rapid degradation in serum. Introduction of chemical modifications to such DsiRNA conjugates to increase nuclease resistance will likely change the interaction of the conjugate with Dicer (Nechaev et al., 2013).

Further, to suppress the expression of the target gene (*STAT3*), researchers conjugated the CpG oligonucleotide with the DNA duplex, which is part of the promoter of the *STAT3* gene, so that when it enters the nucleus of the target cell, this duplex binds to the corresponding transcription factor and blocks its transcription (Sen et al., 2012). This conjugate showed a therapeutic effect in a mouse model of acute myeloid leukemia after several intravenous injections (5 mg/kg) (Zhang et al., 2016). The first stage of clinical trials of this conjugate for the treatment of B-cell non-Hodgkin's lymphoma is planned for 2019.

### Dynamic Polyconjugates

Dynamic polyconjugates, which contain two types of cleavable bonds, were developed by Arrowhead Pharmaceuticals to facilitate the endosomal escape of siRNA (Rozema et al., 2007). A polyconjugate is an amphiphilic polymer consisting of poly-(butyl-aminovinyl ether) (PBAVE), to which polyethylene glycol residues and a targeted ligand molecule (N-acetylgalactosamine) are attached using an acid-cleavable carboxy dimaleimide anhydride linker. siRNA molecules are connected to PBAVE through linkers containing disulfide bonds that can be cleaved in the cytoplasm of the cell. Thus, following penetration of the dynamic polyconjugate by receptor-mediated endocytosis into the cell and entry into the acidic environment of the endosome, the carboxy-diimide anhydrite bonds are cleaved and N-acetylgalactosamine and polyethylene glycol dissociate from the polyconjugate. Following this, the newly formed amino groups on PBAVE are protonated, which leads to a decrease in endosomal pH, an increase in osmotic pressure, and rupture of the endosomal membrane. Conjugates of PBAVE and siRNA are released into the cytoplasm, followed by cleavage of the disulfide bond and detachment of the siRNA from the polymer (Rozema et al., 2007). Due to effective endosomal escape, dynamic polyconjugates exhibit high biological activity: suppression of the target gene *F7* was observed in cynomolgus monkeys with 99% efficiency for 80 days following a single intravenous injection (5 mg/kg) (Rozema et al., 2015). Another drug based on a dynamic polyconjugate is the PBAVE polymer, conjugated with polyethylene glycol or N-acetylgalactosamine residues (NAG-PBAVE), but without covalent attachment of siRNA (Wong et al., 2012). In this case, NAG-PBAVE is injected with cholesterol-modified siRNA. Intravenous injection of the cholesterol-siRNA conjugate results in accumulation of siRNA mainly in the liver; the NAG-PBAVE component also accumulates in this organ. This co-administration increases the biological activity of the cholesterol-siRNA substantially: 75% suppression of the target gene (*ApoB*) in the livers of rhesus monkeys was observed over 30 days following one intravenous injection of the drug (2 mg/kg siRNA and 15 mg/kg NAG-PBAVE) (Wong et al., 2012).

Another drug developed by Arrowhead Pharmaceuticals represents a conjugate of siRNA and cholesterol administered together with a conjugate of N-acetylgalactosamine and melitin-like peptide (NAG-MLP) (Wooddell et al., 2013). Melitin-like protein is an endosomolytic pore-forming peptide capable of increasing the efficiency of the release of the cholesterol conjugate from endosomes and, therefore, its biological activity. It has been shown that after single intravenous injection of the cholesterol-siRNA conjugate (1 mg/kg) together with NAG-MLP (6 mg/kg), expression of the *F7* target gene in the mouse liver was suppressed with 99% efficiency, while the cholesterol-siRNA conjugate (10 mg/kg) alone, without NAG-MLP, reduced expression of the *F7* gene only by 20% (Wooddell et al., 2013). This co-administration system was used to treat chronic hepatitis B virus in patients in clinical trials. A 90% decrease of hepatitis B surface antigen (HBsAg) was observed for 50 days after a single injection of 4 mg/kg of a mixture of two cholesterol-siRNA conjugates (anti-*HBx* and anti-*preC-C*) and NAG-MLP.

However, clinical trials of several drugs based on PBAVE and melitin for the treatment of liver diseases were halted due to high toxicity demonstrated in a non-human primate study (Turner et al., 2018). The company switched to TRiM technology based on the conjugation of siRNA and N-acetylgalactosamine; however, the specific structure of the drug has not yet been disclosed (Wooddell et al., 2017) (see section “siRNA and receptor ligand conjugates”).

## siRNA Conjugates in the Clinic

Onpattro (Patisiran), the first commercially available siRNA-based drug, was released for the treatment of hereditary transthyretin polyneuropathy in August 2018 by Alnylam Pharmaceuticals (Adams et al., 2018; Garber, 2018; Solomon et al., 2019). Onpattro is an anti-*TTR* siRNA containing several 2′O-Me modifications in complex with a cationic lipid, phospholipid, cholesterol, and a conjugate of polyethylene glycol and lipid. Its administration every 3 weeks for 18 months contributes to a significant reduction in the symptoms of the disease compared with patients taking placebo. However, the fact that its use has to be combined with corticosteroids, acetaminophen, and antihistamines is evidence of the pro-inflammatory effect of the lipids used in Onpattro. Moreover, the side effects of this drug include redness, nausea, headache, pain in the back and abdomen, and breathing difficulties. Due to these reasons, subsequent drugs developed by Alnylam Pharmaceuticals do not use lipids for delivery and are presented as conjugates of siRNA and N-acetylgalactosamine. Currently, six drugs based on this platform are at the most advanced steps of development in Alnylam pipline; three are in the third stage, and three are in the second and first stages of clinical trials (Huang, 2017). Moreover, these conjugates all have the same structure and chemical modifications (2′O-Me, 2′F, and PS) and differ only in siRNA sequences and chemical modification patterns.

Advanced products under development by other companies (Dicerna Pharmaceuticals, Arrowhead Pharmaceuticals, and Silence Therapeutics) that suppress gene expression in hepatocytes are based on covalent conjugates of siRNA and N-acetylgalactosamine (Crooke et al., 2018; Nikam and Gore, 2018; Springer and Dowdy, 2018). Thus, significant success was achieved in the delivery of siRNA to liver cells; the search for new targets for siRNA and the determination of the dose required for a therapeutic effect will expand the range of drugs aimed at the liver (Zatsepin et al., 2016; Shen and Corey, 2017). The design of systems for delivery to organs is a fast developing area, however, currently such drugs are only at the preclinical stage (Benizri et al., 2019), successful delivery to such organs as the kidneys may be the next step (Khvorova and Watts, 2017).

## Challenges and Limitations of Using siRNA Bioconjugates in Clinics

The use of chemical modifications in siRNA conjugates significantly improved their bioperformance allowed to solve such problems as: some of non-target effects—the cellular immune response is reduced by the presence of 2′O-Me siRNA modifications (Judge et al., 2006); the probability of RISC^*^ binding to non-target mRNA molecules can be reduced by decreasing the melting temperature of the seed region of the siRNA by introducing UNA or GNA modifications (Janas et al., 2017); the use of a fully modified siRNA molecules increases the time of inhibition of the target gene up to half of the year (Ray et al., 2017). Bioconjugation strategies described above can improve the ability of conjugates to accumulate in certain organs and penetrate certain types of target cells without the help of transfection agents or other means of delivery have been developed. However, there are still a number of unsolved problems that limit the possibility of transfer of drugs from the laboratory bench to the clinic. The main problem of this kind is the low bioavailability of siRNA conjugates and unfavorable pharmacokinetics, which, together with the rather high cost of obtaining such drugs in quantities necessary to achieve a therapeutic effect, impedes their use in the clinic. The low bioavailability of siRNA conjugates is primarily due to the fact that in order for siRNA to enter the cytoplasm of a target cell after systemic administration, it has to overcome numerous barriers—the endothelial barrier when leaving the bloodstream to the tissue, as well as to escape siRNA from the endosome to the cytoplasm. The overcoming of these barriers is complicated by the unfavorable pharmacokinetics of such drugs, associated with their relatively low molecular weight, which lies below the filtration limit, due to which the drugs are rapidly removed from the bloodstream by renal filtration. In this regard, the development of alternative routes of administration, such as subcutaneous or local in which there is a deposit and the gradual release of the drug is promising, as well as approaches aimed at increasing the duration of the circulation of the drug in the bloodstream (Nair et al., 2017). Another option—the targeted delivery, which can be implemented only for some organs and tissues with sufficient efficiency, moreover, using the advantage of specific binding to target cells does not cancel the dependence of this process on the concentration of drug in the blood or interstitial fluid and the duration of maintenance of an effective concentration. The necessity to administer high doses of drugs to achieve a therapeutic effect raises the problem of specificity and possible side effects, the severity of which increases at high concentrations. It can be expected, that along with non-specific effects associated with the suppression of partially homologous targets, which can be eliminated by sequence selection and conjugate design, immunostimulation, the metabolic effects of unnatural analogs, including cumulative and long-term ones, as well as the intervention of exogenous siRNA into cellular regulatory systems of miRNA in competition for RISC, can become the main non-specific effects important for the safety of clinical use. In this regard, the main challengers of biomedical research are increasing the bioavailability, biological activity and targeting of delivery, which will reduce the therapeutic doses of drugs based on siRNA conjugates.

## Conclusions

The introduction of molecules of natural origin into the composition of siRNA is a promising approach for non-viral delivery and has clear advantages over other approaches (physical methods, delivery using cationic lipids, and polymers): specificity of penetration into target cells and absence of toxic effects (Lee et al., 2016; Benizri et al., 2019). The primary difficulty in designing bioconjugates is the necessity of selecting specific ligands for individual applications due to the specificity of ligand-receptor interactions. From this point of view, the use of lipophilic siRNA conjugates is less specific, since LDL receptors are expressed at a high level by various cell types; however, it could be beneficial if high selectivity of delivery to certain cell types is not required and accumulation of the drug into non-target cells does not cause undesirable effects (Turanov et al., 2018). The latest patterns of chemical modifications can reduce the ID_50_ and increase the duration of the biological effect of siRNA conjugates. As a result, the application of siRNA-based drugs in clinical practice in the next few years may significantly increase.

## Author Contributions

IC wrote the manuscript. VV and EC critically analyzed and corrected the text. EC came up with the concept.

### Conflict of Interest Statement

The authors declare that the research was conducted in the absence of any commercial or financial relationships that could be construed as a potential conflict of interest.
