# Supplementary material for: Current Development of siRNA Bioconjugates: From Research to the Clinic
Source: Front Pharmacol. 2019 Apr 26;10:444. doi: 10.3389/fphar.2019.00444 (PMC6498891; doi:10.3389/fphar.2019.00444)
Supplement: Supplementary file 1 [file Table_1.pdf]

**Table 1. Bioconjugates.**

| Conjugate | Structure and modifications[1), 2), 3), 4)] | Target cells/organ and gene | Concentration or dose | Silencing | Ref. |
|---|---|---|---|---|---|
| **Lipophilic siRNA** | | | | | |
| Cholesterol-siRNA 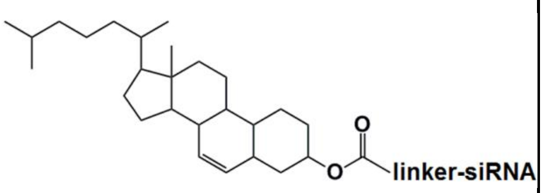 | 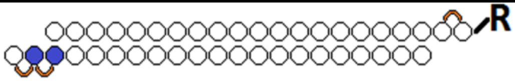 2’O-Me and PS modifications, aminocaproic acid-pyrrolidine linker at 3’ end of S 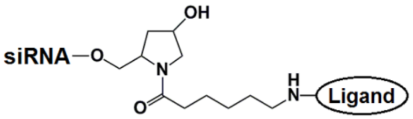 | Mouse liver (1) and jejunum (2), *ApoB* | 50 mg/kg, 3 i.v. injections | 57% (1), 42% (2), mRNA (after 24 h) | (Soutschek et al., 2004) |
| | as in (Soutschek et al., 2004) | Mouse liver (1), *ApoB* | 50 mg/kg, i.v. | 50 % mRNA, (after 48 h) | (Wolfrum et al., 2007) |
| | 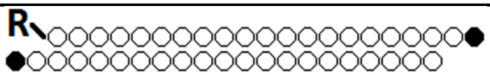 Inverted thymidines, disulfide linker at the 5’ end of S $-(CH_2)_6-S-S-(CH_2)_6-$ | Mouse lungs, *p38* | 7.5 mg/kg, intratracheal injection | 40 % mRNA, (after 12 h) | (Moschos et al., 2007) |
| | No modifications, aminohexyl linker from 5’ end of S | Huh-7 cells, *β-Gal* | 50 nM | 55 % mRNA | (Lorenz et al., 2004) |
| | 11 2’O-Me modifications, no pattern data, aminohexyl linker at 5’ end of S | MCF7 cells, *IRS1* | 200 nM | 50 % protein | (Cesarone et al., 2007) |
| | 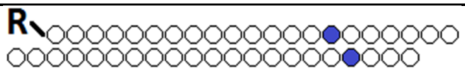 | Xenograft KB-8-5 | 10 mg/kg, i.v. | 60 % protein, | (Chernikov |

| | | | | | |
|---|---|---|---|---|---|
| | 2'O-Me modifications, aminohexyl linker at 5' end of S | tumor in SCID mice, *MDR1* | | (after 6 days) | et al., 2017) |
| | 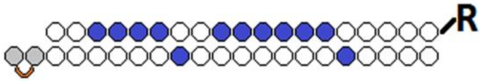 2'O-Me modifications, linker at 3' end of S 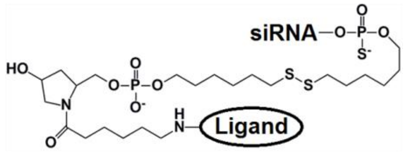 | Rat spinal cord, *CNS* | ~4 mg/kg (injection 10 µl/h, during 3 days) | 60% mRNA (after 24 h) | (Chen et al., 2010) |
| | 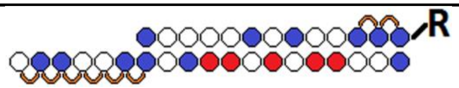 2'O-Me, 2'F and PS modifications, tetraethyleneglycol (TEG) linker at 3' end of S 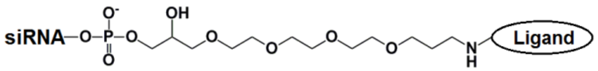 | Rat eye, *Ppib* | 0.15 mg/kg, intraocular injection | 55% mRNA (after 2 days) | (Byrne et al., 2013) |
| | 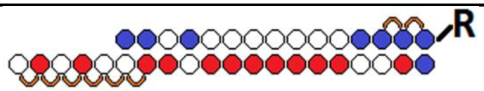 2'O-Me, 2'F and PS modifications, TEG linker at 3' end of S | Mouse brain: striatum (1) and cortex (2), *Htt* | ~1.25 mg/kg, intracranial injection (i.c.) to striatum | 75 % (1), 40 % (2) protein (after 5 days) | (Alterman et al., 2015) |
| | 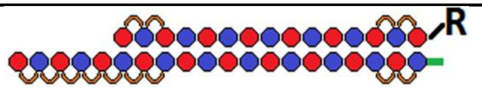 2'O-Me, 2'F, PS and 5'(*E*)-vinylphosphonate modifications, C7 linker at 3' end of S 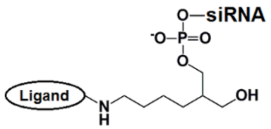 | Mouse liver (1) and kidneys (2), *Htt* and *Ppib* | 20 mg/kg, subcutaneous injection (s.c.) | 50 and 70 % (1), ~5 and 30% (2) mRNA (after 7 days) | (Biscans et al., 2018) |

| | | | | | |
|---|---|---|---|---|---|
| | 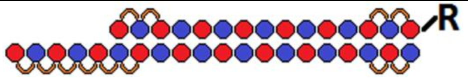<br>2’O-Me, 2’F and PS modifications, TEG linker at 3’ end of S | Placenta of pregnant CD1 mice, *sFLT1* | 20 mg/kg, i.v. | 25 % mRNA (after 5 days) | (Turanov et al., 2018) |
| | 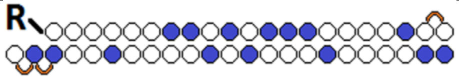<br>2'O-Me and PS modifications, aminohexyl linker at 5’ end of S | Liver of ICR mice, *Factor VII* (*F7*) | 5 mg/kg, i.v. | 0% protein (after 2 days) | (Wong et al., 2012) |
| | 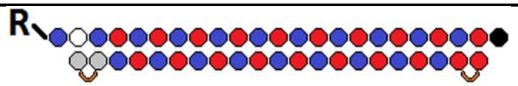<br>2'O-Me, 2'F, dNMP, PS and inverted thymidine, aminohexyl linker at 5’ end of S | Liver of ICR mice, *Factor VII* (*F7*) | 10 mg/kg, i.v. | 20 % protein, (after 2 days) | (Wooddell et al., 2013) |
| Cholesterol-siRNA and polyconjugate (N-acetylgalactosamine-PBAVE) | 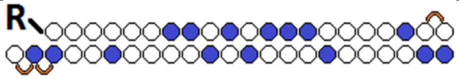<br>2'O-Me and PS modifications, aminohexyl linker at 5’ end of S<br>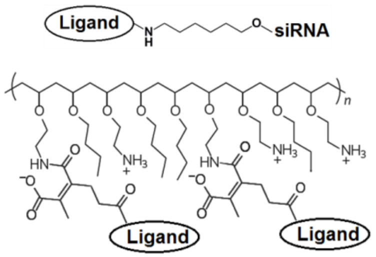 | Rhesus monkey liver, *ApoB* | 2 mg/kg siRNA and 15 mg/kg NAG-PBAVE, i.v. | 75% protein (during 30 days) | (Wong et al., 2012) |
| Cholesterol-siRNA and conjugate of N-acetylgalactosamine and melittin-like peptide (NAG-MLP) | 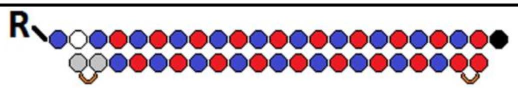<br>2'O-Me, 2'F, dNMP, PS and inverted thymidine, | Liver of cynomolgus monkeys, *Factor VII* (*F7*) | 2 mg/kg siRNA and 3 mg/kg NAG-MLP, i.v. | 99 % protein, (during 25 days) | (Wooddell et al., 2013) |

| | | | | | | |
|---|---|---|---|---|---|---|
| | aminohexyl linker at 5' end of S 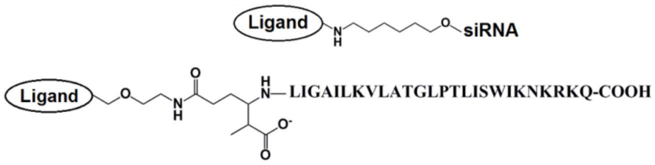  | Human liver, HBV infection, *HBx*, *preC-C* | 4 mg/kg mix of siRNA and NAG-MLP, i.v. | ~90 % HBsAg (Hepatitis B surface Antigen), (during 50 days) | (Wooddell et al., 2017) |
| Docosanyl-siRNA (C22) 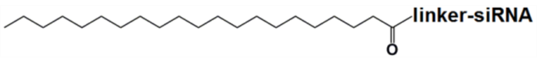  | as in (Soutschek et al., 2004) | Mouse liver, *ApoB* | 50 mg/kg, 3 i.v. injections | 50 % mRNA (after 24 h) | (Wolfrum et al., 2007) |
| Stearoyl-siRNA (C18) 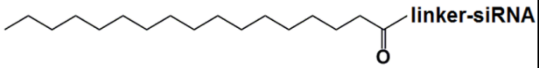  | | | | 30 % mRNA (after 24 h) | |
| Lithocholic-oleyl-siRNA 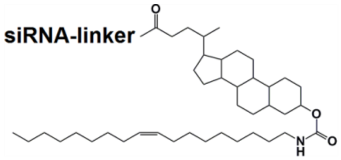  | | | | 35 % mRNA (after 24 h) | |
| Palmitoyl-ssPHK (C16) 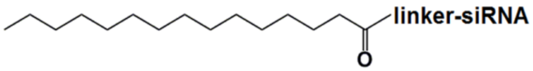  | 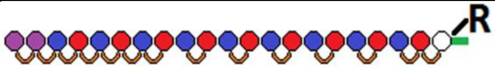  2'O-Me, 2'O-MOE, 2'F, PS and 5'(*E*)- vinylphosphonate modifications single stranded siRNA, aminohexyl linker is attached at 5' end of S through 2' position of the ribose | Liver of transgenic mouse, *ApoC III* (Human gene) | 14 mg/kg, s.c. (IC50~10 mg/kg) | 65% mRNA, (after 2 days) | (Prakash et al., 2015) |
| α-Tocopherol- Dicer-substrate siRNA (DisRNA) 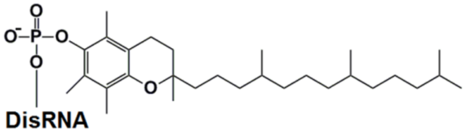  | 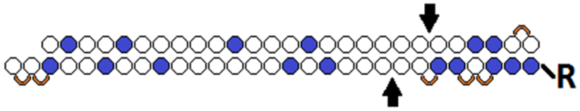  2'O-Me and PS modifications, α-tocopherol is attached at 5' end of AS through 5' | Mouse liver, *ApoB* | 32 mg/kg, i.v. | 80 % mRNA, (after 2 days) ($IC_{50}$ ~2 mg/kg) | (Nishina et al., 2008) |

| | | | | | |
|---|---|---|---|---|---|
| | phosphate[3)] | | | | |
| **Conjugates of siRNA and peptides** | | | | | |
| SPACE-siRNA «ACKTGSHNQCG»-siRNA | All nucleotides are 2’O-Me modified (1), or without modifications (2), amide linker at 3’ end of AS | Mouse epidermis, *IL10* | Application to the skin, 3 $cm^2$, ~12 mg/kg | 23 % (1), 28% (2), protein, (after 24 h) | (Hsu and Mitragotri, 2011) |
| cRGD-siRNA «RGDFK»-siRNA (cyclic peptide) | 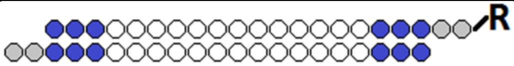 2’O-Me and dNMP modifications, linker at 3’ end of AS 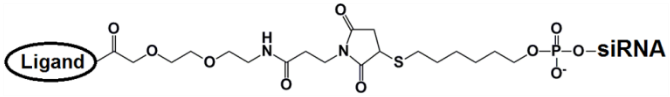 | Xenograft A549 tumor in BALB/c nude mouse, *VEGFR2* | 0.752 mg/kg, 6 i.v. injections every 3 days | 55% mRNA, 60% protein, reduced tumor growth rate | (Liu et al., 2014) |
| TAT-siRNA «YGRKKRRQRRR»-siRNA | No modifications, linker at 3’ end of AS -N-end-Cys-HN-$(CH_2)_3$- | HeLa cells, *GFP* | 400 nM | 60 % protein | (Chiu et al., 2004) |
| | Inverted thymidines, linker at 3’ end of AS -C-end-Cys –S-$(CH_2)_6$- | Mouse lungs, *MAPK14* | 7.5 mg/kg, intratracheal | 40 % mRNA (after 12 h) | (Moschos et al., 2007) |
| TAT-siRNN (neutral ribonucleic acid) | 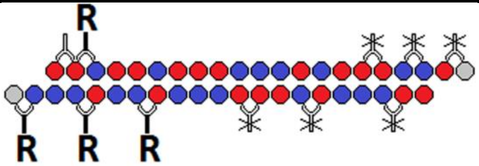 2’O-Me purines, 2’F pyrimidines, dNMP and *t*Bu-SATE modifications, linkers are at phosphates of both strands 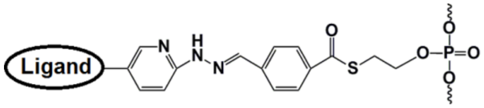 | H1299-dGFP cells, *GFP* | 50 nM | 85 % protein | (Meade et al., 2014) |
| Penetratin-siRNA | No modifications, linker at 5’ end of S | C166-GFP (1), | 25 nM | 80 % (1), | (Muratovs |

| | | | | | |
|---|---|---|---|---|---|
| «CRQIKIWFQNRRMKWKK»-siRNA | -N-end-Cys-S- | EOMA-GFP (2) cells, *GFP* | | 95 % (2) protein | ka and Eccles, 2004) |
| | Inverted thymidines, linker at 5’ end of S<br>-C-end-Cys –S-$(CH_2)_6$- | Lungs of mouse, *MAPK14* | 7.5 mg/kg intratracheal | 20 % mRNA (after 12 h) | (Moschos et al., 2007) |
| Transportan-siRNA «CLIKKALAALAKLNIKLLYGASNLTWG»- siRNA | No modifications, linker at 5’ end of S<br>-N-end-Cys-S- | COS-7 cells, *F. luciferase* | 25 nM | 50 % protein | (Muratovska and Eccles, 2004) |
| | | C166-GFP (1), EOMA-GFP (2) cells, *GFP* | 25 nM | 68 % (1) 97 % (2) protein | |
| «Peptide that is cleaved by caspase 4»-siRNA<br>«LEVDG»-siRNA | No modifications, aminohexyl linker at 5’ end of AS | Jed-3 cells, *STAT3* | 200 nM | 70 % protein | (Koehn et al.) |
| «Peptide analogue of IGF1»-siRNA<br>«CSKC»-siRNA | 11 2’O-Me, no pattern data, aminohexyl linker at 5’ end of S | MCF7 cells, *IRS1* | 200 nM | 65 % protein | (Cesarone et al., 2007) |
| Albumin-siRNA | 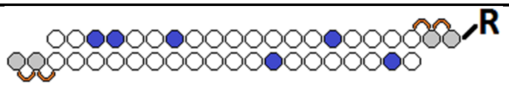  2’O-Me, PS and dNMP modifications, linker at 3’ end of S 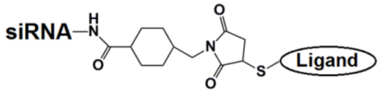  | Heart of BALB/c mouse, *IGF-IR* | 5 mg/kg, i.v. | 35 % mRNA (after 3 days) | (Lau et al., 2012) |
| **N-acetylgalactosamine-conjugated siRNA** | | | | | |

| | | | | | |
|---|---|---|---|---|---|
| N-acetylgalactosamine-siRNA | 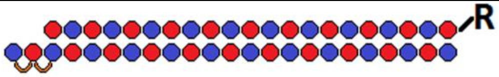 (1) 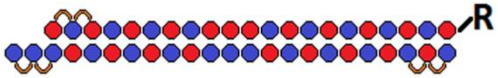 (2)<br>2’O-Me, 2’F and PS modifications, linker at 3’ end of S<br>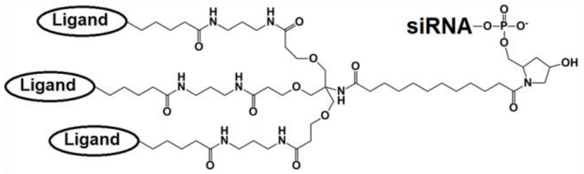 | Mouse liver, *transthyretin* (*TTR*) | 5 mg/kg, s.c. | 55 % (1), 85% (2) mRNA (during more than 40 days) | (Nair et al., 2014) |
| | 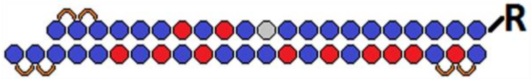<br>2’O-Me, 2’F, PS and dNMP modifications (Advanced ESC), linker as in (Nair et al., 2014) at 3’ end of S | Human liver, *PCSK9* | ~6 mg/kg (500 mg), s.c. | 70 % protein (during 6 months) | (Ray et al., 2017) |
| N-acetylgalactosamine-siRNN | 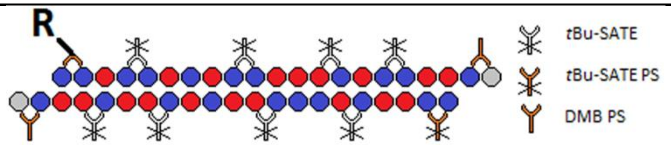<br>2’O-Me, 2’F, *t*Bu-SATE, DMB, PS and dNMP modifications, linker attached to the internucleotide phosphate at 5’ end of S<br>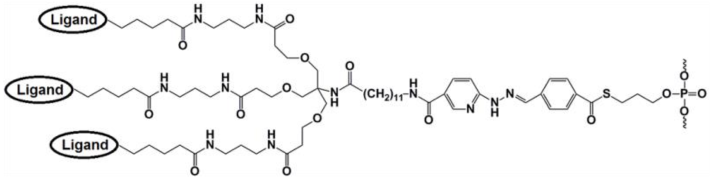 | Mouse liver, *ApoB* | 25 mg/kg, i.v. | 60 % mRNA (during 12 days) | (Meade et al., 2014) |
| **Conjugates of siRNA and aptamers** | | | | | |

| | | | | | |
|---|---|---|---|---|---|
| «RNA aptamer (A10) to PSMA»-siRNA | No modifications,<br>Aptamer is attached through adenine at 5’ end of S[4)] | LNCaP cells, *PLK1* | 400 nM | 85 % mRNA | (McNamara et al., 2006) |
| | | Xenograft LNCaP tumor in athymic mice, *PLK1* | 0.3 mg/kg (200 pmole), 10 intratumoral injections (i.t.) during 20 days | Tumor regression | |
| «RNA aptamer (A10-3) to PSMA»-shPHK | No modifications,<br>Aptamer is attached through the uracil at 5’ end of S[4)] | LNCaP cells, *PRKDC* | 400 nM | 85 % mRNA | (Ni et al., 2011) |
| | | Xenograft LNCaP tumor in nude mice, *PRKDC* | 0.3 mg/kg (200 pmole), 2 i.t. injections, together with ionizing radiation | 65 % mRNA, tumor regression | |
| «RNA aptamer (A-1) to gp120»- DisRNA | 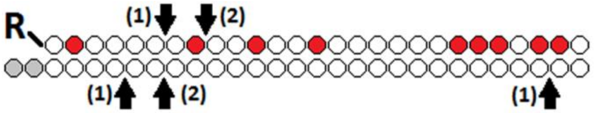<br>2’F pyrimidines, aptamer is attached through two uracils at 5’ end of S[4)] | HIV-1 infection in humanized $Rag2^{-/-}$ $\gamma c^{-/-}$ mice, *Tat*, *Rev* | 0.38 mg/kg (250 pmole), 8 i.v. injections during 4 weeks | 90 % mRNA (at PBMC), reduction of HIV-1 particles in plasma by $10^5$ times (during 4 weeks) | (Neff et al., 2011) |
| «RNA aptamer (Gint4.T) to beta-type platelet-derived growth factor receptor (PDGFRβ)»-siRNA | 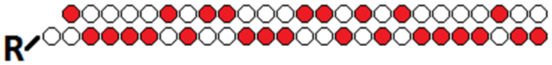<br>2’F modifications, linker is the duplex containing 2’O-Me and 2’F modifications and $(CH_2)_3$, complementarily | Xenograft U87MG tumor in NOD/SCID nude mice, *STAT3* | 2.6 mg/kg (1.6 nmole), 5 intrapetitally injections (i.p.) | 60 % mRNA; 77 % protein (after 3 days), tumor growth | (Esposito et al., 2018) |

| | | | | | |
|---|---|---|---|---|---|
| | binds to 3' end of the AS 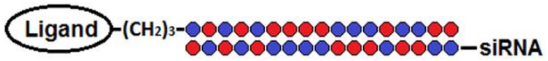 | | for 9 days | retardation | |
| «DNA aptamer (aptNCL) to nucleolin»-siRNA | The DNA aptamer has two poly-T tails attached through a sulfhydryl group to the sulfosuccinimidyl-4- (N-maleimidophenyl) butyrate at 3′ end of S | Xenograft CL1-5 tumor in NOD-SCID mice, *SLUG*, *NPR1* | 0.1 mg/kg (50 pmole), 15 i.t. injections for 5 weeks | 95 % protein, tumor growth retardation | (Lai et al., 2014) |
| **Conjugates of siRNA and antibodies** | | | | | |
| «Ab to insulin receptor»-siRNA | No modifications, biotin-streptavidin linker at 5’ end of S | HEK 293 cells, *F. luciferase* | 115 nM | 90 % protein | (Xia et al., 2009) |
| «AT (hu3S193) to Lewis-Y»-siRNA | dNMP modifications, linker at 5’ end of AS 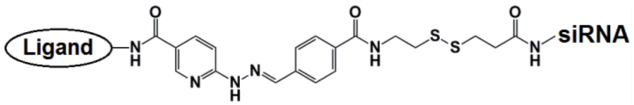 | A431 cells, *STAT3* | 300 nM (1), 300 nM and 100 µM chloroquine (2) | 0 % (1), 60 % (2), mRNA | (Ma et al., 2011) |
| «Ab (TfRMAb) to transferrin receptor»-siRNA | No modifications, TEG biotin-streptavidin linker at 5’ end of S | Gliobastoma RG-2 cells implanted in the brain in CD344 rats, *F. luciferase* | 0.27 mg/kg, i.v. | 81 % protein (after 2 days) | (Xia et al., 2007) |
| «$F_{ab}$ fragment (RI7 217) to CD71»-siRNA | 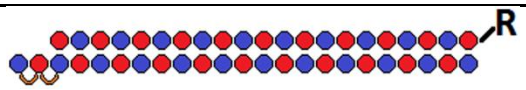 2’O-Me, 2’F and PS modifications, linker at 3’ end of S 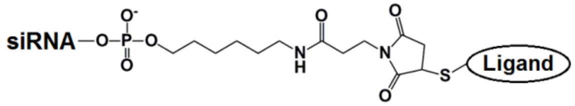 | Peripheral artery disease model in C57BL/6J mice, *MSTN* | ~3.6 mg/kg, 4 intramuscular injections for 4 weeks | 72 % mRNA, increase the running distance by 24 % | (Sugo et al., 2016) |
| «Ab to TENB2»-siRNA | 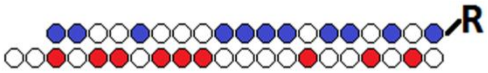 | Xenograft PC3-TENB2-high tumor in | 24 mg/kg, 3 i.v. injections | ~33% mRNA, (after 2 days) | (Cuellar et al., 2014) |

| | | | | | |
|---|---|---|---|---|---|
| | 2’O-Me and 2’F modifications, linker as in (Lau et al., 2012) at 3' end of S | nude mice, *PPIB* | | | |
| **Other siRNA conjugates** | | | | | |
| CpG-DisRNA TCCATGACGTTCCTGATGCT-DisRNA | No modifications, C15 hydrocarbon linker at 5' end of AS | AML model, *Cbfb-MYH11/Mpl*$^{+}$ cells i.v. injected in *NOD/SCID/IL-2RγKO* mice, *STAT3* | 5 mg/kg, 6 i.v. injections for 6 days | 45% mRNA, in AML cells (after 24 h) | (Hossain et al., 2014) |
| Dynamic polyconjugate | 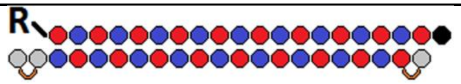  2'O-Me, 2'F, dNMP, PS and inverted thymidine, linker at 5' end of S 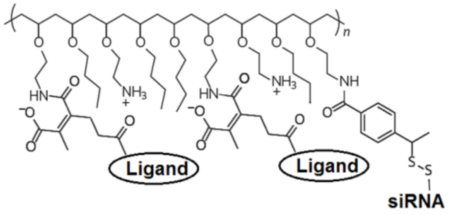  | Liver of cynomolgus monkeys, *Factor VII* (*F7*) | 5 mg/kg, i.v. | 99 % protein, (during 80 days) | (Rozema et al., 2015) |

1) sense strand (S) 3’-5’ is above, antisense strand (AS) 3’-5’ is below;

2) white color indicates unmodified RNA nucleotides; 2’O-Me is blue; 2’F is red; dNMP is grey; PS is orange; inverted thymidine is black; 2′O-MOE is violet; 5’(*E*)- vinylphosphonate is green;

3) “R” is a linker with ligand;

4) The estimated cleavage sites by the Dicer are indicated by arrows.
